# White Matter Abnormalities and Cognition in Aging and Alzheimer Disease

**DOI:** 10.1001/jamaneurol.2025.1601

**Published:** 2025-06-09

**Authors:** Christopher Peter, Aditi Sathe, Niranjana Shashikumar, Kimberly R. Pechman, Abigail W. Workmeister, T. Bryan Jackson, Yuankai Huo, Shubhabrata Mukherjee, Jesse Mez, Logan C. Dumitrescu, Katherine A. Gifford, Corey J. Bolton, Leslie S. Gaynor, Shannon L. Risacher, Lori L. Beason-Held, Yang An, Konstantinos Arfanakis, Guray Erus, Christos Davatzikos, Duygu Tosun-Turgut, Mohamad Habes, Di Wang, Arthur W. Toga, Paul M. Thompson, Panpan Zhang, Kurt G. Schilling, Marilyn Albert, Walter Kukull, Sarah A. Biber, Bennett A. Landman, Barbara B. Bendlin, Sterling C. Johnson, Julie Schneider, Lisa L. Barnes, David A. Bennett, Angela L. Jefferson, Susan M. Resnick, Andrew J. Saykin, Paul K. Crane, Michael L. Cuccaro, Timothy J. Hohman, Derek B. Archer, and the Alzheimer’s Disease Sequencing Project Phenotype Harmonization Consortium (ADSP-PHC) Analyst Team, Dimitrios Zaras, Yisu Yang, Alaina Durant, Praitayini Kanakaraj, Michael E. Kim, Chenyu Gao, Nancy R. Newlin, Karthik Ramadass, Nazirah Mohd Khairi, Zhiyuan Li, Tianyuan Yao, Seo-Eun Choi, Brandon Klinedinst, Michael L. Lee, Phoebe Scollard, Emily H. Trittschuh, Elizabeth A. Sanders

**Affiliations:** 1 Vanderbilt Memory and Alzheimer’s Center, Vanderbilt University School of Medicine, Nashville, Tennessee; 2 Department of Computer Science, Vanderbilt University, Nashville, Tennessee; 3 Department of Electrical and Computer Engineering, Vanderbilt University, Nashville, Tennessee; 4 Department of Medicine, University of Washington, Seattle; 5 Department of Neurology, Boston University Chobanian and Avedisian School of Medicine, Boston, Massachusetts; 6 Department of Neurology, Vanderbilt University Medical Center, Nashville, Tennessee; 7 Vanderbilt Genetics Institute, Vanderbilt University Medical Center, Nashville, Tennessee; 8 Vanderbilt Brain Institute, Vanderbilt University Medical Center, Nashville, Tennessee; 9 Department of Radiology and Imaging Sciences, Indiana University School of Medicine, Indianapolis; 10 Indiana Alzheimer’s Disease Research Center, Indiana University School of Medicine, Indianapolis; 11 Laboratory of Behavioral Neuroscience, National Institute on Aging, National Institutes of Health, Baltimore, Maryland; 12 Department of Biomedical Engineering, Illinois Institute of Technology, Chicago; 13 Rush Alzheimer’s Disease Center, Rush University Medical Center, Chicago, Illinois; 14 Department of Diagnostic Radiology, Rush University Medical Center, Chicago, Illinois; 15 Department of Radiology, University of Pennsylvania, Philadelphia; 16 Department of Radiology and Biomedical Imaging, University of California, San Francisco; 17 Glenn Biggs Institute for Alzheimer’s and Neurodegenerative Diseases, University of Texas Health Science Center at San Antonio, San Antonio; 18 University of Texas Health Science Center at San Antonio, San Antonio; 19 Laboratory of Neuroimaging, Mark and Mary Stevens Neuroimaging and Informatics Institute, Keck School of Medicine, University of Southern California, Los Angeles; 20 Imaging Genetics Center, Mark and Mary Stevens Neuroimaging and Informatics Institute, Keck School of Medicine, University of Southern California, Marina del Rey; 21 Department of Biostatistics, Vanderbilt University Medical Center, Nashville, Tennessee; 22 Department of Radiology and Radiological Sciences, Vanderbilt University Medical Center, Nashville, Tennessee; 23 Vanderbilt University Institute of Imaging Science, Vanderbilt University Medical Center, Nashville, Tennessee; 24 Department of Neurology, Johns Hopkins School of Medicine, Baltimore, Maryland; 25 National Alzheimer’s Coordinating Center, University of Washington, Seattle; 26 Department of Biomedical Engineering, Vanderbilt University, Nashville, Tennessee; 27 Wisconsin Alzheimer’s Disease Research Center, School of Medicine and Public Health, University of Wisconsin, Madison; 28 Wisconsin Alzheimer’s Institute, School of Medicine and Public Health, University of Wisconsin–Madison, Madison; 29 Rush Alzheimer’s Disease Center, Rush University Medical Center, Chicago, Illinois; 30 John P. Hussman Institute for Human Genomics, University of Miami, Miami, Florida; 31 Dr John T. MacDonald Foundation Department of Human Genetics, University of Miami, Miami, Florida; 32 Department of Psychiatry and Behavioral Sciences, University of Washington School of Medicine, Seattle; 33 Geriatric Research Education and Clinical Center, Veterans Affairs Puget Sound Healthcare System, Seattle, Washington

## Abstract

**Importance:**

There has yet to be a large-scale study quantifying the association between white matter microstructure and cognitive performance and decline in aging and Alzheimer disease (AD).

**Objective:**

To investigate the associations between tract-specific white matter microstructure and cognitive performance and decline in aging and AD-related cognitive impairment.

**Design, Setting, and Participants:**

This prognostic study of aging and AD, a secondary data analysis of multisite cohort studies, acquired data from 9 cohorts between September 2002 and November 2022. Participants were eligible if they had diffusion-weighted magnetic resonance imaging (dMRI) data, domain-specific cognitive composite *z* scores, demographic and clinical data, were aged 50 years or older, and passed neuroimaging quality control. Demographic and clinical covariates included age, sex, education, race and ethnicity, *APOE* haplotype status (ε2, ε3, ε4), and clinical status. The present study was conducted from June 2024 to February 2025.

**Exposures:**

White matter microstructure and cognitive performance and decline.

**Main Outcomes and Measures:**

Clinical diagnosis, imaging measures (dMRI, T1-weighted MRI, and amyloid and tau positron emission tomography), and cognitive tests.

**Results:**

Of 4467 participants who underwent 9208 longitudinal cognitive sessions, 2698 (60.4%) were female, and the mean age (SD) was 74.3 (9.2) years; 3213 were cognitively unimpaired, 972 had mild cognitive impairment, and 282 had AD dementia. White matter free water (FW) showed the strongest associations with cross-sectional cognitive performance and longitudinal cognitive decline across all domains, particularly memory. FW in limbic tracts, such as the cingulum, presented the strongest associations with both memory performance (cingulum: β = −0.718; *P* < .001; fornix: β = −1.069; *P* < .001) and decline (cingulum: β = −0.115; *P* < .001; fornix: β = −0.153; *P* < .001). White matter FW measures interacted with baseline diagnosis, gray matter atrophy, *APOE* ε4 status, and amyloid positivity to predict poorer cognitive performance and accelerated cognitive decline. Noteworthy interactions include fornix FW and hippocampal volume (β = 10.598; *P* < .001), cingulum FW and SPARE-AD index (β = −0.532; *P* < .001), and inferior temporal gyrus transcallosal tract FW and baseline diagnosis (β = −0.537; *P* < .001), all predicting poorer memory performance.

**Conclusions and Relevance:**

White matter microstructural changes, particularly FW, play a critical role in cognitive decline in aging and AD-related cognitive impairment. These findings highlight the importance of FW correction in dMRI studies and highlight the limbic system, especially the cingulum and fornix, as key regions associated with cognitive decline; the interaction models highlight that integrating FW-corrected metrics with other AD biomarkers may further elucidate the biological mechanisms of neurodegeneration in aging.

## Key Points

**Question:**

What is the association between white matter microstructure and cognitive performance and decline in aging and Alzheimer disease (AD)?

**Findings:**

In this large, multicohort prognostic study of 4467 participants and 9208 longitudinal cognitive sessions, white matter free water was associated with cognitive performance and decline, especially memory. Limbic tracts (eg, cingulum, fornix) were most strongly associated across domains, and abnormal AD endophenotypes (eg, SPARE-AD index, *APOE *ε4 status, amyloid positivity) exacerbated cognitive decline.

**Meaning:**

White matter microstructural changes were associated with cognitive decline, emphasizing their role in aging and AD assessment; interaction models support a multimodal approach to improve the prediction of cognitive decline.

## Teaser

This prognostic study investigates the associations between tract-specific white matter microstructure and cognitive performance and decline in aging- and Alzheimer disease–related cognitive impairment.

## Introduction

Alzheimer disease (AD) is a progressive neurodegenerative disorder characterized by neuritic plaques and neurofibrillary tangles.^1^ AD is the most common form of dementia, affecting approximately 1 in 9 people (10.9%) aged 65 years and older in the United States.^2^ Numerous studies have demonstrated the utility of diffusion-weighted magnetic resonance imaging (dMRI) in characterizing the neuroanatomical correlates of distinct cognitive profiles along the AD clinical continuum.^3,4^ Research using diffusion tensor imaging has highlighted the role of white matter abnormalities in AD.^5^ However, conventional diffusion tensor imaging metrics (fractional anisotropy, mean diffusivity, axial diffusivity, radial diffusivity) are limited by partial volume effects. Bi-tensor models address this by separating diffusion properties of brain tissue from surrounding free water (FW), a metric posited to be associated with neuroinflammation and atrophy, while FW-corrected intracellular metrics reflect tissue damage, such as demyelination and axonal degeneration.^6,7^ Multishell dMRI is traditionally considered more accurate for quantifying FW,^8^ but its longer acquisition time and advanced processing requirements limit clinical feasibility. Single-shell dMRI, however, enables faster data acquisition and analysis of legacy datasets, allowing for broader participant inclusion and greater statistical power. Established methods have shown they can reliably estimate FW using bi-tensor modeling on single-shell dMRI acquisitions.^6^ These metrics have been successfully applied to a large spectrum of neurodegenerative disorders, including Parkinson disease,^9,10,11^ stroke,^12^ essential tremor,^13^ schizophrenia,^14^ and AD.^4,15,16,17,18^

To date, most neuroimaging research has focused on gray matter atrophy in addition to amyloid and tau associations in AD^19^; however, examining white matter microstructure and cognitive decline may enhance our understanding of neurodegeneration in normal aging and AD. Our group recently found FW measures to be sensitively associated with episodic memory and executive function, both cross-sectionally and longitudinally.^4^ Additionally, we found that these measures interacted with gray matter atrophy to predict more rapid rates of cognitive decline, suggesting that a multimodal approach may enhance our ability to predict cognitive decline.

To our knowledge, no comprehensive study has yet quantified the association between white matter microstructure and cognitive performance and decline in a large-scale dataset. Advances like longitudinal ComBat^20^ and cognitive cocalibration^21^ now enable harmonization of dMRI data and cognitive measures across cohorts, facilitating large-scale analyses. This study leverages these methods to examine associations between white matter microstructure and cognitive domains (memory, executive function, language) in 4467 participants across 9208 cognitive visits. Using single-shell FW correction, we quantified FW and FW-corrected metrics (fractional anisotropy, axial diffusivity, radial diffusivity, and mean diffusivity) in 48 tracts. We hypothesized that tract-specific abnormalities would correlate with cognitive performance and that these abnormalities, interacting with key AD measures (eg, gray matter atrophy, amyloid and tau positivity), would predict accelerated cognitive decline.

## Methods

### Study Cohorts

In this prognostic cohort study, we collated a multisite dataset using participants from 9 cohorts of cognitively unimpaired individuals and cognitively impaired individuals (with mild cognitive impairment and dementia due to AD) between September 2002 and November 2022: the Alzheimer’s Disease Neuroimaging Initiative (ADNI); Baltimore Longitudinal Study of Aging (BLSA); Biomarkers of Cognitive Decline Among Normal Adults (BIOCARD); National Alzheimer’s Coordinating Center (NACC); a combined cohort from the Religious Orders Study (ROS), Rush Memory and Aging Project (MAP), and Minority Aging Research Study (MARS); Vanderbilt Memory and Aging Project (VMAP); and Wisconsin Registry for Alzheimer’s Prevention (WRAP) (eMethods in Supplement 1). Each cohort required demographic and clinical covariates, including age, sex, educational attainment, self-reported race and ethnicity, *APOE* haplotype status, and cognitive status (cognitively unimpaired, mild cognitive impairment, dementia due to AD), with cohort-specific inclusion and exclusion criteria. Additional cohort details can be found in the eMethods in Supplement 1. The current study was conducted from June 2024 to February 2025. Participants were included in this study if they had dMRI, cognitive composite, demographic, and clinical data, were aged 50 years or older, and passed neuroimaging quality control. All participants provided written informed consent, and research was conducted under institutional review board–approved protocols. The Vanderbilt University Medical Center Institutional Review Board approved secondary analysis of these data, and we followed the Strengthening the Reporting of Observational Studies in Epidemiology (STROBE) reporting guidelines.

### Cognitive Composites

Neuropsychological data were independently collected for each cohort and harmonized using established procedures.^21^ Experts assigned test items to memory, executive function, language, or visuospatial domains or none of these. Investigators ensured consistent scoring of anchor items—tests were administered and scored identically across cohorts. Anchor items had identical parameters across studies, while parameters for other items (nonanchor and nonidentical candidate anchor items) were freely estimated in confirmatory bifactor models. Parameters from all available items were used to score each participant at each time point, and *z*-score cognitive composites were created. Full details on all cognitive composites can be found in the original study by Mukherjee et al.^21^ The current study focused on cross-sectional and longitudinal memory, executive function, and language performance.

### dMRI Preprocessing

All data were preprocessed using the PreQual pipeline, which is an automated pipeline that corrects dMRI data for susceptibility-induced motion, geometric distortion, and eddy current effect-induced artifacts, performs intervolume registration to alleviate participant head motion, denoises, and imputes signal dropout in a slicewise manner.^22,23,24,25^ Sessions passing quality control served as inputs for custom MATLAB code (MathWorks) to calculate FW and FW-corrected maps, including FW, FW-corrected fractional anisotropy, FW-corrected axial diffusivity, FW-corrected radial diffusivity, and FW-corrected mean diffusivity. A standard space representation was created by nonlinearly warping the conventional fractional anisotropy map to the FMRIB58_FA atlas using symmetric normalization and linear interpolation in the Advanced Normalization Tools package.^26^ Participants with age-regressed outliers (±5 SD) in FW or FW-corrected metrics were excluded, and all reported sample sizes account for these exclusions.

### White Matter Tracts of Interest and Microstructural Harmonization

All tractography templates used in this study were drawn from existing resources^4,27,28,29,30,31^ and are available in a publicly available Zenodo repository.^32^ These templates cover transcallosal (TC) projections of the prefrontal, motor, parietal, and occipital tracts in addition to association, projection, and limbic tracts. We quantified mean FW and FW-corrected microstructure within all tracts of interest for each imaging session, totaling 240 tract-specific microstructural measures (5 microstructural measures × 48 tracts). These values were input into longitudinal ComBat, which controlled for imaging batch effects while preserving variance due to several cohort-specific differences, including age, age^2^, sex, diagnosis (at baseline), age × converter interactions, and age^2^ × converter interactions. A random-effects variable was used to control for participant-specific white matter trajectories. Imaging batch grouping details can be found in eTable 1 in Supplement 1. Although we input our entire in-house longitudinal dataset (number of participants = 5144; number of imaging sessions = 10 346) into the harmonized procedures to increase statistical power, the current study was limited to the subset of participants with cognitive data.

### Statistical Analysis

All statistical analyses were performed in R version 4.1.0 statistical software (R Foundation). Age was scaled and centered for all analyses. To assess covariate differences between studies, we first evaluated normality using the Shapiro-Wilk test. For normally distributed continuous variables, we tested for homogeneity of variances using the Levene test and performed analysis of variance (ANOVA) when variances were equal; otherwise, Welch ANOVA was used. For nonnormal distributions, the Kruskal-Wallis test was applied. For categorical variables, we used the χ^2^ test, opting for Fisher exact test when expected frequencies were low.

We first examined cross-sectional data with cognitive performance, leveraging data from 4467 unique participants. Linear regressions were conducted separately for each tract of interest and microstructural variable combination, using scaled values to obtain standardized β coefficients. Covariates included age, education, sex, self-reported race and ethnicity, clinical status at baseline, and *APOE* ε4 and *APOE* ε2 carrier status. In the secondary analysis, we assessed associations between baseline white matter microstructure and longitudinal cognitive performance (ie, cognitive decline) using linear mixed-effects regression. This analysis included participants with multiple cognitive assessments: memory (n = 2307; 7048 sessions), executive function (n = 2301; 7031 sessions), and language (n = 2297; 7014 sessions). These models included the same covariates plus *interval*, *baseline age × interval*, and *white matter at baseline × interval* terms, together with random aging effects for each participant. Interval was defined as the current age minus the age at baseline. Summary statistics were extracted from the *white matter at baseline × interval* term.

To identify white matter microstructural measures most sensitive to cognitive decline, we performed bootstrapped head-to-head comparisons (n = 1000 per tract × measure combination) to compare the marginal variance added beyond a covariate-only model. ANOVAs identified the microstructural metrics, tract types (eg, limbic, occipital TC), and specific tracts contributing the most variance. All analyses were corrected for multiple comparisons using the false discovery rate.

Significant main associations were followed by white matter interactions with diagnosis and *APOE* ε4 status. We also integrated gray matter (hippocampal volume,^19^ Spatial Pattern of Abnormalities for Recognition of Alzheimer’s Disease [SPARE-AD] index^33^) and amyloid^34^ and tau^35,36^ positron emission tomography (PET) measures from the most recent Alzheimer’s Disease Sequencing Project Phenotype Harmonization Consortium (ADSP-PHC) data release. Gray matter analyses included 2975 participants (5384 sessions), while amyloid and tau PET analyses included 693 and 434 participants, respectively. Detailed methods for the ADSP-PHC data are available at https://dss.niagads.org/.

## Results

### Demographic Characteristics

**Figure 1.**
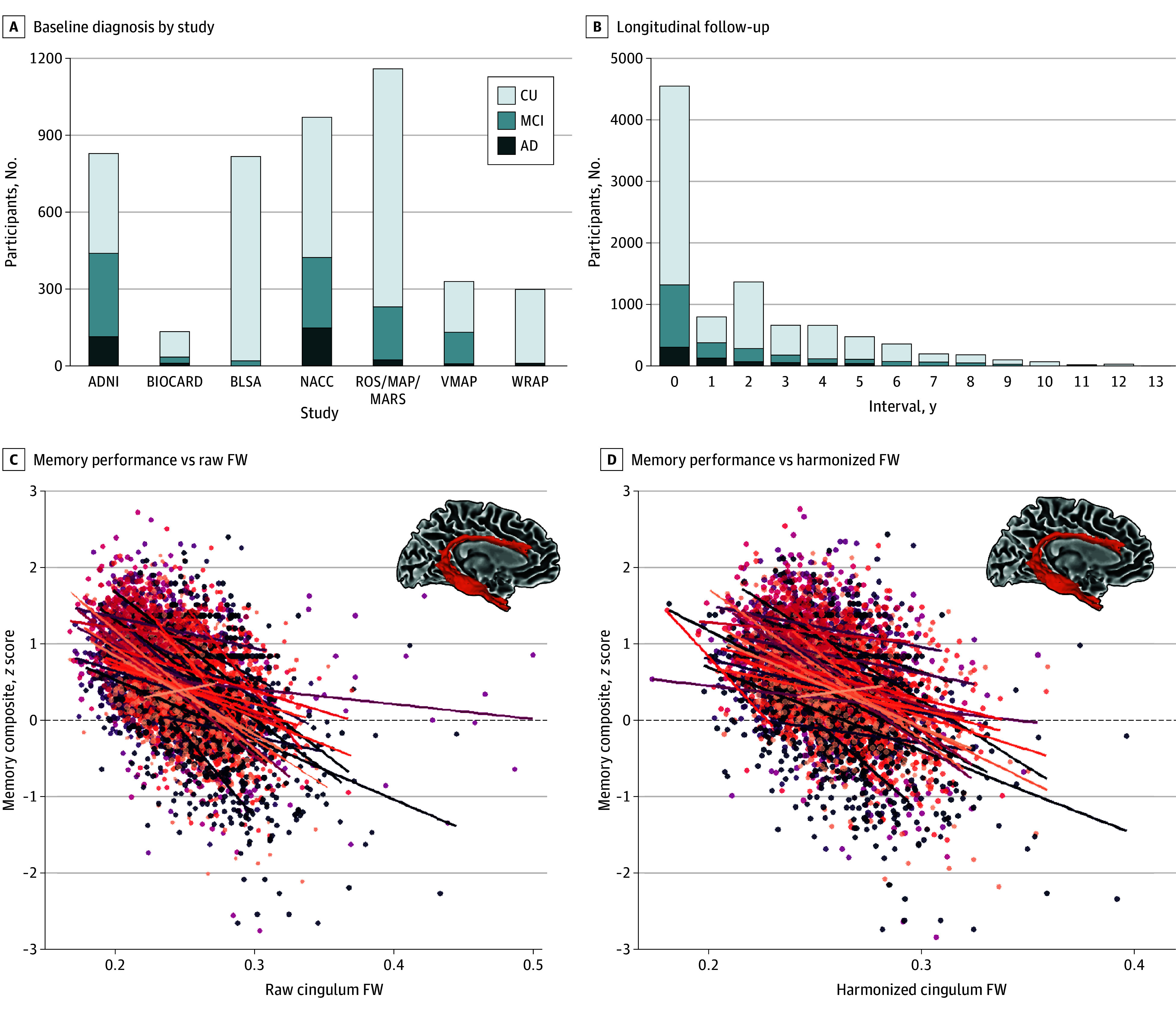
Cohort Characteristics and Data Harmonization A, Participants were drawn from 9 well-established cohorts, including 3213 cognitively unimpaired (CU) individuals, 972 with mild cognitive impairment (MCI), and 282 with Alzheimer disease (AD) at baseline. B, The study also incorporated longitudinal data across 9208 cognitive sessions, spanning up to 13 years of follow-up. Longitudinal ComBat harmonization was applied to all imaging features to account for variability across imaging batches. C and D, Associations are shown between cingulum free-water (FW) and memory performance, using both raw (C) and harmonized (D) FW data, with points and lines color coded by imaging batch. Harmonized data were used across all analyses. ADNI indicates Alzheimer’s Disease Neuroimaging Initiative; BIOCARD, Biomarkers of Cognitive Decline Among Normal Adults; BLSA, Baltimore Longitudinal Study of Aging; MAP, Rush Memory and Aging Project; MARS, Minority Aging Research Study; NACC, National Alzheimer’s Coordinating Center; ROS, Religious Orders Study; VMAP, Vanderbilt Memory and Aging Project; WRAP, Wisconsin Registry for Alzheimer’s Prevention.

The Table provides an overview of the sample sizes, demographic information, clinical status characteristics, and domain-specific cognitive composites at baseline. Of the 4467 participants who underwent 9208 longitudinal cognitive sessions, 2698 (60.4%) were female, and the mean age (SD) was 74.3 (9.2) years; 3213 were cognitively unimpaired, 972 had mild cognitive impairment, and 282 had AD dementia. All demographic and clinical variables differed between cohorts, which was not surprising given our large sample size—particularly notable differences included baseline clinical status, follow-up duration, and baseline age. Figure 1A illustrates the baseline clinical status breakdown for each cohort, and Figure 1B shows the follow-up characteristics in our dataset. Figure 1C and D illustrate the raw and harmonized cingulum bundle FW associations with memory performance. Cohort characteristics for our gray matter, amyloid PET, and tau PET interaction analyses can be found in eTables 2 through 4 in Supplement 1.

**Table.** Participant Characteristics by Dataset

Characteristic	Cohort							
	ADNI	BLSA	BIOCARD	NACC	ROS/MAP/MARS	VMAP	WRAP	Total
Participants, No.	830	757	127	974	1160	326	293	4467
Sessions, No.	1801	1936	148	1305	2558	1031	429	9208
Visits, mean (SD), No.	2.17 (1.53)	2.56 (1.63)	1.17 (0.37)	1.34 (0.64)	2.21 (1.38)	3.16 (1.09)	1.46 (0.69)	2.06 (1.37)
Follow-up time, mean (SD), y	2.68 (2.13)	3.98 (2.16)	1.96 (0.29)	1.65 (0.89)	4.28 (2.50)	2.98 (1.40)	3.47 (1.77)	3.47 (2.24)
Age at baseline, mean (SD), y	74.75 (7.50)	70.45 (10.28)	71.81 (7.40)	74.95 (8.11)	79.48 (7.32)	73.32 (7.23)	62.01 (6.20)	74.27 (9.18)
Sex, No. (%)								
Female	413 (49.8)	418 (55.2)	79 (62.2)	563 (57.8)	896 (77.2)	135 (41.4)	194 (66.2)	2698 (60.4)
Male	417 (50.2)	339 (44.8)	48 (37.8)	411 (42.2)	264 (22.8)	191 (58.6)	99 (33.8)	1769 (39.6)
Education, mean (SD), y	16.33 (2.59)	17.00 (2.39)	17.44 (2.19)	14.51 (3.96)	15.84 (3.31)	15.83 (2.67)	16.62 (2.78)	15.94 (3.22)
*APOE* ε4 carrier, No. (%)	296 (35.7)	193 (25.5)	47 (37.0)	385 (39.5)	220 (19.0)	115 (35.3)	90 (30.7)	1346 (30.1)
Baseline clinical status, No. (%)								
CU	393 (47.4)	749 (98.9)	102 (80.3)	550 (56.5)	936 (80.7)	194 (59.5)	289 (98.6)	3213 (71.9)
MCI	325 (39.2)	6 (0.8)	22 (17.3)	276 (28.3)	209 (18.0)	131 (40.2)	3 (1.0)	972 (21.8)
AD	112 (13.5)	2 (0.3)	3 (2.4)	148 (15.2)	15 (1.3)	1 (0.3)	1 (0.3)	282 (6.3)
Diagnosis converters, No. (%)^a^	468 (56.4)	40 (5.3)	31 (24.4)	445 (45.7)	327 (28.2)	163 (50.0)	4 (1.4)	1478 (33.1)
Composite *z* score at baseline, mean (SD)								
Memory composite	0.49 (0.86)	0.48 (0.47)	1.27 (0.49)	0.24 (0.87)	0.39 (0.53)	0.44 (0.55)	1.21 (0.41)	0.47 (0.72)
Executive function composite	0.45 (0.67)	0.40 (0.38)	0.94 (0.38)	0.09 (0.79)	0.54 (0.55)	0.09 (0.50)	0.87 (0.32)	0.40 (0.64)
Language composite	0.49 (0.64)	0.50 (0.44)	0.99 (0.56)	0.38 (0.74)	0.41 (0.58)	0.56 (0.53)	1.13 (0.34)	0.51 (0.62)

Abbreviations: AD, Alzheimer disease; ADNI, Alzheimer’s Disease Neuroimaging Initiative; BLSA, Baltimore Longitudinal Study of Aging; BIOCARD, Biomarkers of Cognitive Decline Among Normal Adults; CU, cognitively unimpaired; MAP, Rush Memory and Aging Project; MARS, Minority Aging Research Study; MCI, mild cognitive impairment; NACC, National Alzheimer’s Coordinating Center; ROS, Religious Orders Study; VMAP, Vanderbilt Memory and Aging Project; WRAP, Wisconsin Registry for Alzheimer’s Prevention.

^a^ Diagnosis converters were defined as individuals who had a non–cognitively unimpaired diagnosis at any longitudinal time point.

### Association of White Matter Microstructure With Cross-Sectional Cognitive Performance

**Figure 2.**
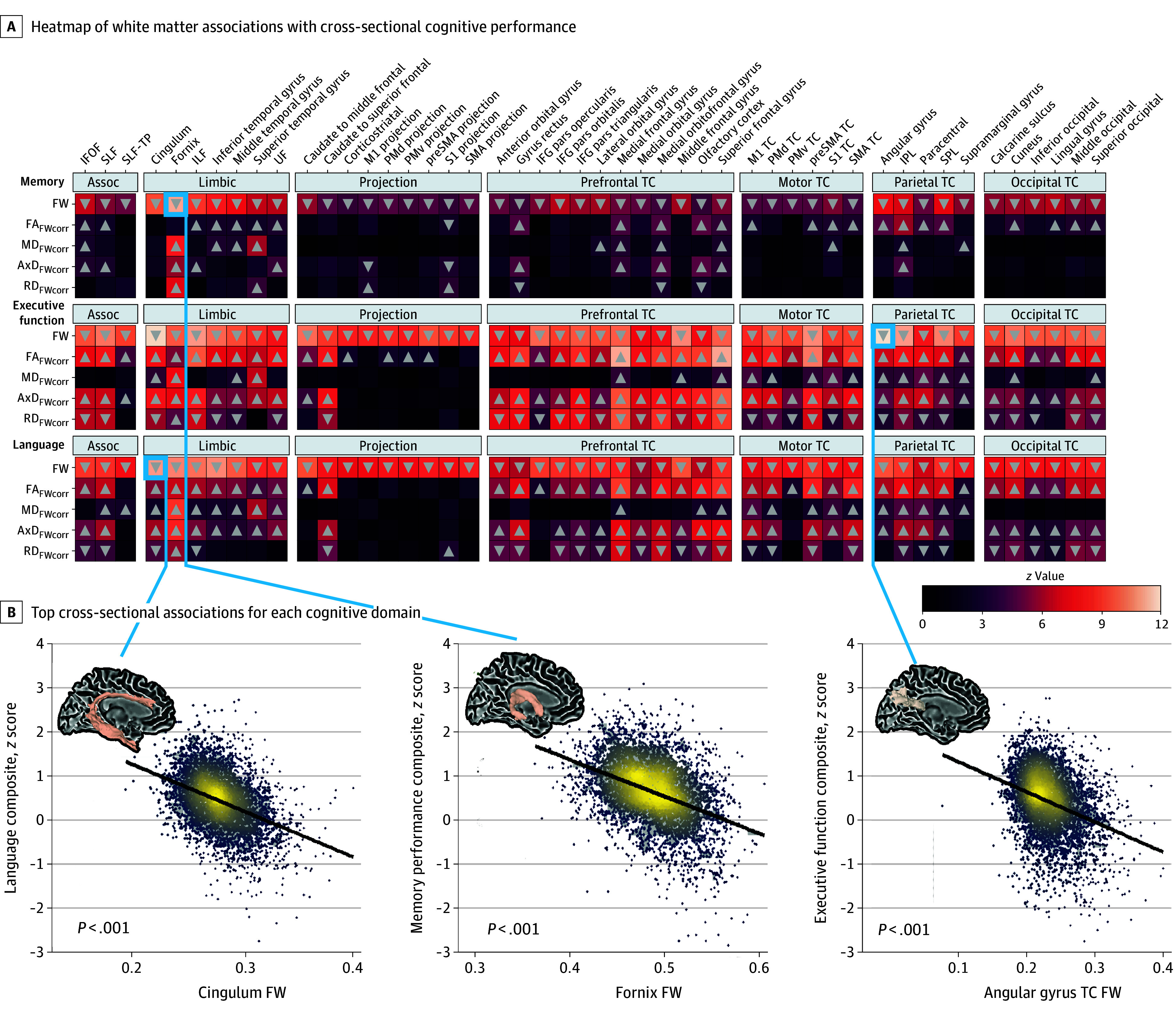
White Matter Association With Cognitive Performance A, Associations are shown between free water (FW)–corrected metrics and baseline cognitive performance across the all-memory (top), executive function (middle), and language (bottom) domains. Linear regression models were conducted for each FW-corrected metric (FW, FW-corrected fractional anisotropy [FA_FWcorr_], FW-corrected mean diffusivity [MD_FWcorr_], FW-corrected axial diffusivity [AxD_FWcorr_], FW-corrected radial diffusivity [RD_FWcorr_]). Each heatmap is grouped by tract type and represents the individual *z* value for each independent model. The arrows represent the direction of β coefficients that reached significance following correction for multiple comparisons. B, The regression plots show the correlations between cognitive performance and the top microstructural association for each domain. Assoc indicates association; IFG, inferior frontal gyrus; IFOF, inferior frontal occipital fasciculus; ILF, inferior longitudinal fasciculus; IPL, inferior parietal lobule; PMd, dorsal premotor cortex; PMv, ventral premotor cortex; preSMA, pre–supplementary motor area; SLF, superior longitudinal fasciculus; SLF-TP, superior longitudinal fasciculus temporal parietal component; SMA, supplementary motor area; SPL, superior parietal lobule; TC, transcallosal; UF, uncinate fasciculus.

We found that higher global FW was associated with lower performance across all cognitive domains (Figure 2). For memory (Figure 2A, top panel), we found global associations (with high limbic involvement), with the top associations being with fornix FW (β = −1.069; *P* < .001) (Figure 2B) and cingulum (β = −0.718; *P* < .001). For executive function (Figure 2A, middle panel), we also found global associations, with the top association being with TC angular gyrus (β = −0.512; *P* < .001) (Figure 2B). For language (Figure 2A, bottom panel), we found global associations, with the top association being with cingulum FW (β = −0.776; *P* < .001). Findings for all cross-sectional analyses can be found in eTable 5 in Supplement 2. Expanded cross-sectional illustrations can be found in eFigure 1 in Supplement 1.

### Association of Baseline White Matter Microstructure With Cognitive Decline

**Figure 3.**
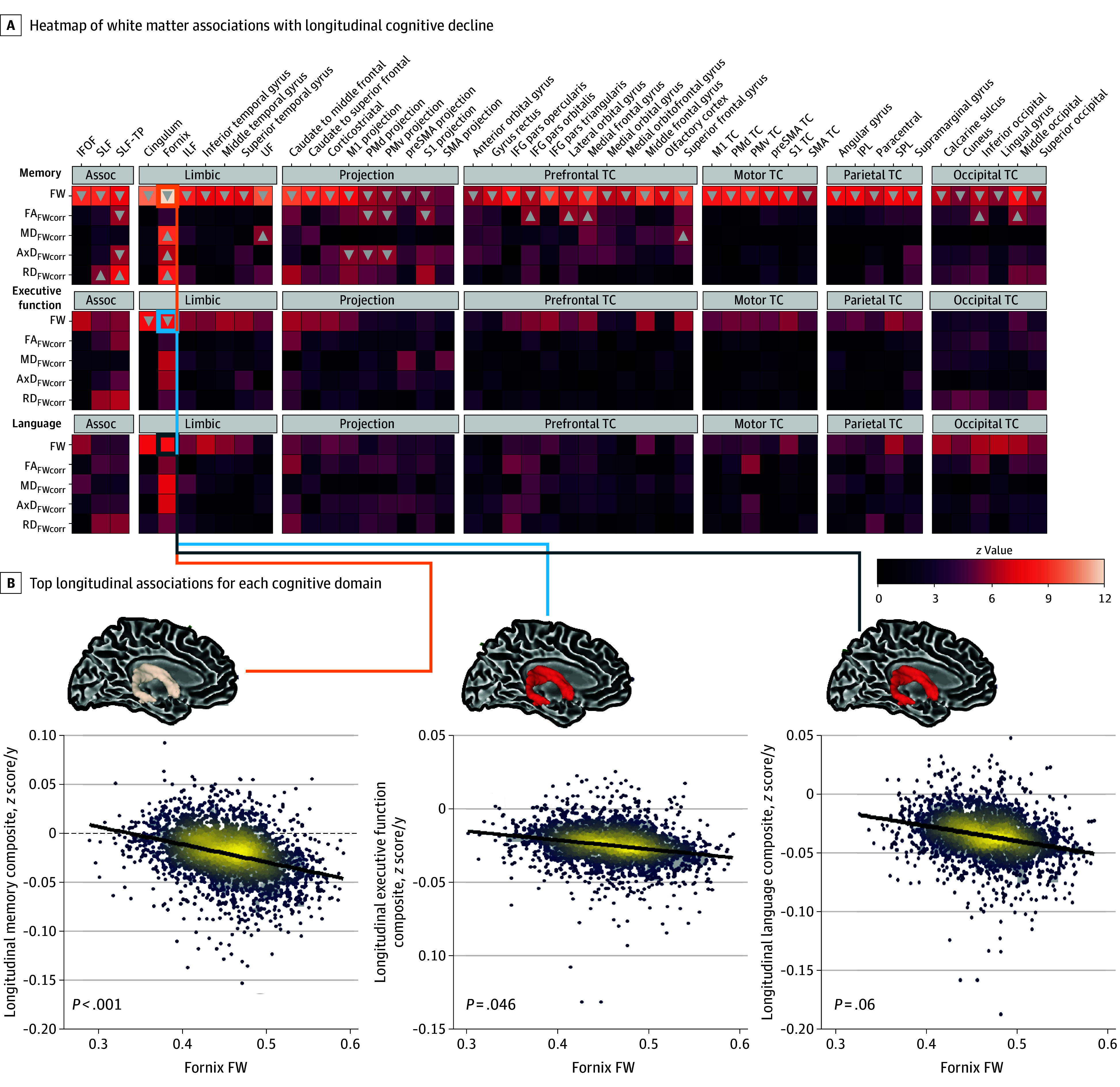
White Matter Association With Cognitive Decline A, Associations are shown between free water (FW)–corrected metrics and longitudinal cognitive decline across the all-memory (top), executive function (middle), and language (bottom) domains. Linear mixed-effects regression models were conducted for each FW-corrected metric (FW, FW-corrected fractional anisotropy [FA_FWcorr_], FW-corrected mean diffusivity [MD_FWcorr_], FW-corrected axial diffusivity [AxD_FWcorr_], FW-corrected radial diffusivity [RD_FWcorr_]). Each heatmap is grouped by tract type and represents the individual *z* value for each independent model. The arrows represent the direction of β coefficients that reached significance following correction for multiple comparisons. B, The regression plots show the correlations between cognitive decline and the top microstructural association for each domain. Assoc indicates association; IFG, inferior frontal gyrus; IFOF, inferior frontal occipital fasciculus; ILF, inferior longitudinal fasciculus; IPL, inferior parietal lobule; PMd, dorsal premotor cortex; PMv, ventral premotor cortex; preSMA, pre–supplementary motor area; SLF, superior longitudinal fasciculus; SLF-TP, superior longitudinal fasciculus temporal parietal component; SMA, supplementary motor area; SPL, superior parietal lobule; TC, transcallosal; UF, uncinate fasciculus.

The longitudinal analysis (Figure 3; eTable 6 in Supplement 2) showed baseline global FW was strongly associated with memory decline, particularly in the fornix (β = –0.153; *P* < .001) and cingulum (β = −0.115; *P* < .001). For executive function, only cingulum (β = −0.073; *P* = .046) and fornix (β = −0.085; *P* = .046) FW showed significant associations, while no significant associations were found for language. Expanded longitudinal illustrations can be found in eFigure 2 in Supplement 1.

### Bootstrapped Head-to-Head Comparisons of White Matter Features Most Associated With Cognitive Decline

**Figure 4.**
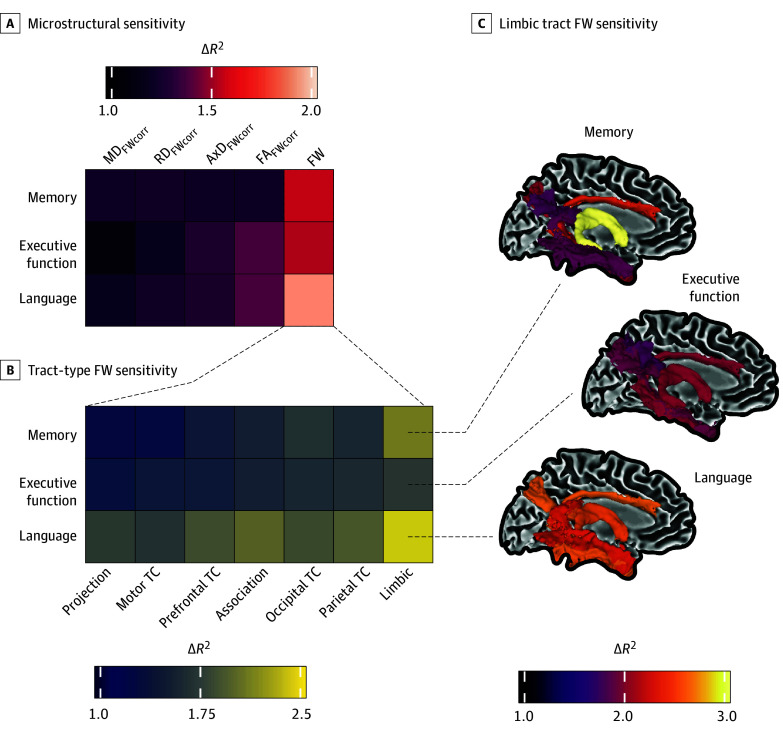
Bootstrapped Head-to-Head Analysis to Compare Microstructure, Tract Type, and Individual Tracts on Cognitive Decline A, Bootstrapped linear mixed-effects regression (n = 1000) identified free water (FW) as the microstructural metric most associated with cognitive decline. B, Within FW, limbic tracts showed the strongest association with cognitive decline. C, Further analysis revealed specific limbic tracts driving this association. Δ*R*^2^ represents the contribution of white matter changes (microstructure and covariates) vs covariates alone. AxD_FWcorr_ indicates FW-corrected axial diffusivity; FA_FWcorr_, FW-corrected fractional anisotropy; MD_FWcorr_, FW-corrected mean diffusivity; RD_FWcorr_, FW-corrected radial diffusivity; TC, transcallosal.

We aimed to identify which microstructural metric was most associated with cognitive decline. First, we conducted a bootstrapped analysis to determine the mean marginal *R*^2^ (in percentage) for a covariate-only model for memory (*R*^2^ = 34.93; 95% CI, 32.06-37.79), executive function (*R*^2^ = 29.30; 95% CI, 26.68-32.18), and language (*R*^2^ = 27.76; 95% CI, 25.05-30.27). FW explained the greatest additional variance (in percentage) as compared with the base (covariates-only) model for memory (Δ*R*^2^ = 1.55; 95% CI, 0.04-3.27), executive function (Δ*R*^2^ = 1.52; 95% CI, 0.14-2.95), and language decline (Δ*R*^2^ = 1.90; 95% CI, 0.51-3.42) (Figure 4A). Results from the microstructural ANOVAs, post hoc *t* tests, and bootstrapped means are provided in eTables 7 through 9 in Supplement 2. A follow-up tract-type analysis (Figure 4B), grouping FW into projection, motor TC, prefrontal TC, association, occipital TC, parietal TC, and limbic tracts, showed that limbic tracts provided the highest additional variance compared with the covariate-only models for decline in memory (Δ*R*^2^ = 2.07; 95% CI, 0.41-4.04), executive function (Δ*R*^2^ = 1.71; 95% CI, 0.31-3.17), and language (Δ*R*^2^ = 2.37; 95% CI, 0.91-3.86). All tract-type findings are provided in eTables 10 through 12 in Supplement 2. Last, Figure 4C highlights the top 5 limbic tracts most associated with cognitive decline, and full results can be found in eTables 13 through 15 in Supplement 2.

### Interaction of White Matter Microstructure With AD Endophenotypes

Our cross-sectional and longitudinal interaction analyses revealed a strong, statistically significant synergistic association between white matter abnormalities and other AD endophenotypes (eTable 16 in Supplement 2) on cognitive performance and decline. In total, we conducted 2826 cross-sectional interaction analyses, with the strongest interactions observed for fornix FW and hippocampal volume (β = 10.598; *P* < .001), cingulum bundle FW and SPARE-AD index (β = −0.532; *P* < .001), and inferior temporal gyrus TC tract FW and baseline diagnosis (β = −0.537; *P* < .001), all predicting poorer memory performance. Longitudinally, we found evidence that white matter abnormalities interact with the SPARE-AD index to predict more rapid cognitive decline. Key results for interactions with *APOE* ε4 positivity, hippocampal volume, SPARE-AD index, and amyloid positivity are highlighted in eFigures 3 through 5 in Supplement 1.

## Discussion

This study leveraged a longitudinal, multisite dataset to examine associations between tract-specific white matter microstructure and cognitive performance and decline in aging and AD. Using dMRI data from 4467 participants across 9208 sessions, we generated FW-corrected maps for 5 microstructural metrics and quantified mean values within 48 white matter tracts, harmonizing these values using longitudinal ComBat. FW consistently explained the greatest variance in cognitive decline across all domains, with limbic tracts, particularly the cingulum and fornix, showing the strongest associations with memory and other domains. Post hoc interaction analyses revealed significant synergistic associations between white matter abnormalities and key AD endophenotypes, including hippocampal volume, SPARE-AD index, *APOE* ε4 positivity, and amyloid PET positivity, in predicting poorer memory and accelerated cognitive decline. These findings emphasize the critical role of FW and its interactions with AD biomarkers in understanding cognitive aging and disease progression.

Our cross-sectional analysis found that FW exhibited global associations with cognitive performance along the cognitive impairment spectrum, consistent with previous findings of widespread white matter degeneration in cognitive aging.^5,16,17,37,38,39,40,41,42,43,44^ Notably, while higher FW was consistently associated with lower performance across all cognitive domains, intracellular metrics showed limited associations with memory but were broadly significant for executive function and language, excluding sensorimotor projection tracts. This suggests that FW, potentially reflecting neuroinflammation or atrophy,^6,7^ may serve as a broader indicator of cognitive decline, whereas intracellular changes, potentially reflecting overt tissue abnormalities, appear to play more domain-specific roles, particularly in executive function and language. Future studies integrating FW measures with genetic data could help clarify the underlying biological mechanisms associated with these metrics.

The strongest FW associations with cognitive decline were found in the cingulum and fornix, particularly in association with memory, which supports our previous work on the high vulnerability of these tracts in both normal and abnormal aging,^16^ especially along the AD diagnosis continuum.^17^ While previous studies found associations between FW-corrected metrics and memory, this study is to our knowledge the first large-scale examination of FW-corrected metric associations with executive function and language. Our findings emphasize the relevance of FW, while also highlighting the role of intracellular metrics for these domains. For executive function, strong intracellular associations were observed within prefrontal and parietal TC tracts, aligning with findings on the role of the prefrontal cortex and its connections, including the middle frontal gyrus, in executive function.^45,46^ For language, intracellular metrics across almost all brain regions (excluding projection tracts) were associated, with top FW-corrected fractional anisotropy associations involving TC tracts from the medial and superior frontal gyri, which show lower functional connectivity in AD and are associated with language performance.^47^ Tracts projecting from the supplementary motor area, essential for speech processing,^48^ also showed significant associations.

Our longitudinal analysis revealed that FW was the most significant microstructural measure associated with cognitive decline across the memory, executive function, and language domains. This aligns with previous studies across the aging and neurodegeneration spectrum, which identify FW as a sensitive neurodegenerative biomarker.^4,7,9,10,11,12,13,14,16,17,49,50^ Further evaluation of FW by tract type highlighted the limbic tracts as being most predictive of subsequent cognitive decline, consistent with the established role of limbic structures in memory and emotion regulation. Specifically, the cingulum and fornix were strongly associated with memory decline, echoing findings that these tracts are vulnerable in aging.^16,17^ Overall, our findings suggest that microstructure in the fornix and cingulum may be valuable biomarkers for aging and AD, with targeted focus on these regions offering potential for more effective interventions to support healthy aging.

This study represents the largest effort to date to pair FW-corrected metrics with other AD endophenotypes to examine how these biomarkers are collectively associated with cognitive performance and decline. Cross-sectionally, we observed strong associations between *APOE* ε4 status, baseline diagnosis, hippocampal volume, and the SPARE-AD index with all cognitive domains. Amyloid positivity, however, was selectively associated with executive function performance. Notably, there were no significant tau positivity interactions after correction for multiple comparisons, although uncorrected *P* values suggest a potential interaction of occipital and limbic FW with executive function performance. Longitudinally, our findings revealed significant interactions between white matter abnormalities and hippocampal volume, baseline diagnosis, SPARE-AD index, and *APOE* ε4 status, particularly in predicting memory decline over time. Interestingly, our hippocampal volume interactions were primarily observed in limbic tracts, whereas SPARE-AD index interactions extended to both limbic and prefrontal tracts, consistent with the default mode network’s involvement in the SPARE-AD index.^33^ Further analysis indicated that baseline diagnosis was most strongly associated with memory performance, followed by language and executive function. While memory is typically the first domain to exhibit measurable changes, prior research suggests that these changes do not always correspond directly with brain abnormalities. Our findings indicate that different biomarkers demonstrate varying sensitivities when paired with white matter metrics to predict domain-specific cognitive changes.^51^ Future research leveraging more comprehensive longitudinal data may be necessary to understand these complex temporal associations.

### Strengths and Limitations

This study has several notable strengths, including the use of a harmonized, multisite dMRI cohort with a sample size exceeding previous FW studies on aging and AD. The inclusion of 48 publicly accessible tractography templates supports comprehensive analyses and enhances reproducibility for future research. However, this study has several limitations. It did not evaluate asymmetry in white matter changes, which may be particularly relevant to the language domain.^37^ Generalizability may be limited by the high representation of well-educated, non-Hispanic White participants, and cohort ascertainment variability introduces potential confounds. Additionally, while FW has been associated with vascular pathologies in prior research,^38^ vascular factors were not controlled in this study and warrant further investigation. The reliance on single-shell FW correction,^6^ although suitable for large-scale analyses, lacks the precision of multishell techniques like neurite orientation dispersion and density imaging.^8^ Last, while cocalibrated cognitive *z* scores^21^ enabled robust cross-study comparisons across memory, executive function, and language domains, it limited the ability to examine item-level associations within each domain.

## Conclusions

This study is, to our knowledge, the first to evaluate the association between FW-corrected white matter metrics and cognitive performance and decline using a large-scale harmonized dataset. Our findings reveal significant associations across all cognitive domains, with memory showing the strongest associations, particularly when interacting with other AD endophenotypes to predict accelerated cognitive decline.

## Notes

Supplement 1. **eMethods.** Supplemental Methods
eReferences
**eTable 1.** Batch Groupings in Longitudinal ComBat of Our Main Free-Water Database **eTable 2.** Participants in Gray Matter x White Matter Interaction Analysis **eTable 3.** Participants in Amyloid Status x White Matter Interaction Analysis **eTable 4.** Participants in Tau Status x White Matter Interaction Analysis **eFigure 1.** White Matter Associations With Cognitive Performance **eFigure 2.** White Matter Associations With Cognitive Decline **eFigure 3.** White Matter Interactions With APOE-ε4 on Memory Performance **eFigure 4.** White Matter Interactions With Gray Matter Atrophy on Memory Performance **eFigure 5.** White Matter Interactions With PET-Derived Amyloid Positivity on Executive Function Performance

Supplement 2. **eTable 5.** Cross-Sectional Results for All Tracts and Microstructural Metrics for Memory, Executive Function, and Language Performance **eTable 6.** Longitudinal Results for All Tracts and Microstructural Metrics for Memory, Executive Function, and Language Decline **eTable 7.** ANOVA Results From Microstructural Bootstrapped Analysis **eTable 8.** Post Hoc *t* Tests for Microstructural ANOVA **eTable 9.** Bootstrapped Means for Microstructural Analysis **eTable 10.** ANOVA Results From Tract-Type Bootstrapped Analysis **eTable 11.** Post Hoc *t* Tests for Tract-Type ANOVA **eTable 12.** Bootstrapped Means for Tract-Type Analysis **eTable 13.** ANOVA Results From Tract-Specific Bootstrapped Analysis **eTable 14.** Post Hoc *t* Tests for Tract-Specific ANOVA **eTable 15.** Bootstrapped Means for Tract-Specific Analysis **eTable 16.** All Cross-Sectional and Longitudinal Interaction Results for All Tracts and Microstructural Metrics for Memory, Executive Function, and Language Performance

Supplement 3. Members of the Alzheimer’s Disease Neuroimaging Initiative (ADNI), Biomarkers of Cognitive Decline Among Normal Adults (BIOCARD) Study Team, Alzheimer's Disease Sequencing Project (ADSP)

Supplement 4. Data Sharing Statement

## References

[1] Jack CR Jr, Knopman DS, Jagust WJ, . Tracking pathophysiological processes in Alzheimer’s disease: an updated hypothetical model of dynamic biomarkers. Lancet Neurol. 2013;12(2):207-216. doi:10.1016/S1474-4422(12)70291-0 23332364 PMC3622225

[2] Alzheimer’s Association. 2024 Alzheimer’s disease facts and figures. Alzheimers Dement. 2024;20(5):3708-3821. doi:10.1002/alz.13809 38689398 PMC11095490

[3] Talwar P, Kushwaha S, Chaturvedi M, Mahajan V. Systematic review of different neuroimaging correlates in mild cognitive impairment and Alzheimer’s disease. Clin Neuroradiol. 2021;31(4):953-967. doi:10.1007/s00062-021-01057-7 34297137

[4] Archer DB, Moore EE, Shashikumar N, . Free-water metrics in medial temporal lobe white matter tract projections relate to longitudinal cognitive decline. Neurobiol Aging. 2020;94:15-23. doi:10.1016/j.neurobiolaging.2020.05.001 32502831 PMC7483422

[5] Stricker NH, Schweinsburg BC, Delano-Wood L, . Decreased white matter integrity in late-myelinating fiber pathways in Alzheimer’s disease supports retrogenesis. Neuroimage. 2009;45(1):10-16. doi:10.1016/j.neuroimage.2008.11.027 19100839 PMC2782417

[6] Pasternak O, Sochen N, Gur Y, Intrator N, Assaf Y. Free water elimination and mapping from diffusion MRI. Magn Reson Med. 2009;62(3):717-730. doi:10.1002/mrm.22055 19623619

[7] Hoy AR, Ly M, Carlsson CM, . Microstructural white matter alterations in preclinical Alzheimer’s disease detected using free water elimination diffusion tensor imaging. PLoS One. 2017;12(3):e0173982. doi:10.1371/journal.pone.0173982 28291839 PMC5349685

[8] Zhang H, Schneider T, Wheeler-Kingshott CA, Alexander DC. NODDI: practical in vivo neurite orientation dispersion and density imaging of the human brain. Neuroimage. 2012;61(4):1000-1016. doi:10.1016/j.neuroimage.2012.03.072 22484410

[9] Burciu RG, Ofori E, Archer DB, . Progression marker of Parkinson’s disease: a 4-year multi-site imaging study. Brain. 2017;140(8):2183-2192. doi:10.1093/brain/awx146 28899020 PMC6057495

[10] Ofori E, Pasternak O, Planetta PJ, . Longitudinal changes in free-water within the substantia nigra of Parkinson’s disease. Brain. 2015;138(pt 8):2322-2331. doi:10.1093/brain/awv136 25981960 PMC4840947

[11] Yang J, Archer DB, Burciu RG, . Multimodal dopaminergic and free-water imaging in Parkinson’s disease. Parkinsonism Relat Disord. 2019;62:10-15. doi:10.1016/j.parkreldis.2019.01.007 30639168 PMC6589363

[12] Archer DB, Patten C, Coombes SA. Free-water and free-water corrected fractional anisotropy in primary and premotor corticospinal tracts in chronic stroke. Hum Brain Mapp. 2017;38(9):4546-4562. doi:10.1002/hbm.23681 28590584 PMC6866851

[13] Archer DB, Coombes SA, Chu WT, . A widespread visually-sensitive functional network relates to symptoms in essential tremor. Brain. 2018;141(2):472-485. doi:10.1093/brain/awx338 29293948 PMC5815566

[14] Carreira Figueiredo I, Borgan F, Pasternak O, Turkheimer FE, Howes OD. White-matter free-water diffusion MRI in schizophrenia: a systematic review and meta-analysis. Neuropsychopharmacology. 2022;47(7):1413-1420. doi:10.1038/s41386-022-01272-x 35034098 PMC9117206

[15] Sathe A, Yang Y, Schilling KG, . Free-water: a promising structural biomarker for cognitive decline in aging and mild cognitive impairment. Imaging Neurosci (Camb). 2024;2:1-16. doi:10.1162/imag_a_00293 39512598 PMC11540062

[16] Archer DB, Schilling K, Shashikumar N, ; Alzheimer’s Disease Neuroimaging Initiative. Leveraging longitudinal diffusion MRI data to quantify differences in white matter microstructural decline in normal and abnormal aging. Alzheimers Dement (Amst). 2023;15(4):e12468. doi:10.1002/dad2.12468 37780863 PMC10540270

[17] Yang Y, Schilling K, Shashikumar N, ; Alzheimer’s Disease Neuroimaging Initiative. White matter microstructural metrics are sensitively associated with clinical staging in Alzheimer’s disease. Alzheimers Dement (Amst). 2023;15(2):e12425. doi:10.1002/dad2.12425 37213219 PMC10192723

[18] Peterson A, Sathe A, Zaras D, ; Alzheimer’s Disease Neuroimaging Initiative (ADNI); BIOCARD Study Team; Alzheimer’s Disease Sequencing Project (ADSP). Sex and *APOE* ε4 allele differences in longitudinal white matter microstructure in multiple cohorts of aging and Alzheimer’s disease. Alzheimers Dement. 2025;21(1):e14343. doi:10.1002/alz.14343 39711105 PMC11781133

[19] Jack CR Jr, Bennett DA, Blennow K, . NIA-AA Research Framework: toward a biological definition of Alzheimer’s disease. Alzheimers Dement. 2018;14(4):535-562. doi:10.1016/j.jalz.2018.02.018 29653606 PMC5958625

[20] Beer JC, Tustison NJ, Cook PA, ; Alzheimer’s Disease Neuroimaging Initiative. Longitudinal ComBat: a method for harmonizing longitudinal multi-scanner imaging data. Neuroimage. 2020;220:117129. doi:10.1016/j.neuroimage.2020.117129 32640273 PMC7605103

[21] Mukherjee S, Choi SE, Lee ML, . Cognitive domain harmonization and cocalibration in studies of older adults. Neuropsychology. 2023;37(4):409-423. doi:10.1037/neu0000835 35925737 PMC9898463

[22] Kim ME, Ramadass K, Gao C, . Scalable, reproducible, and cost-effective processing of large-scale medical imaging datasets. Proc SPIE. 2025;13411:1341100. doi:10.1117/12.3047273 PMC12734404 41450588

[23] Cai LY, Yang Q, Hansen CB, . PreQual: an automated pipeline for integrated preprocessing and quality assurance of diffusion weighted MRI images. Magn Reson Med. 2021;86(1):456-470. doi:10.1002/mrm.28678 33533094 PMC8387107

[24] Schilling KG, Blaber J, Huo Y, . Synthesized b0 for diffusion distortion correction (Synb0-DisCo). Magn Reson Imaging. 2019;64:62-70. doi:10.1016/j.mri.2019.05.008 31075422 PMC6834894

[25] Kim M, Gao C, Ramadass K, ; Alzheimer’s Disease Neuroimaging Initiative; HABSHD Study Team. Scalable quality control on processing of large diffusion-weighted and structural magnetic resonance imaging datasets. arXiv. Preprint posted online September 24, 2024. 10.1371/journal.pone.0327388 PMC12316263 40748971

[26] Avants BB, Epstein CL, Grossman M, Gee JC. Symmetric diffeomorphic image registration with cross-correlation: evaluating automated labeling of elderly and neurodegenerative brain. Med Image Anal. 2008;12(1):26-41. doi:10.1016/j.media.2007.06.004 17659998 PMC2276735

[27] Archer DB, Bricker JT, Chu WT, . Development and validation of the automated imaging differentiation in parkinsonism (AID-P): a multicentre machine learning study. Lancet Digit Health. 2019;1(5):e222-e231. doi:10.1016/S2589-7500(19)30105-0 33323270

[28] Archer DB, Coombes SA, McFarland NR, DeKosky ST, Vaillancourt DE. Development of a transcallosal tractography template and its application to dementia. Neuroimage. 2019;200:302-312. doi:10.1016/j.neuroimage.2019.06.065 31260838 PMC6703915

[29] Archer DB, Vaillancourt DE, Coombes SA. A template and probabilistic atlas of the human sensorimotor tracts using diffusion MRI. Cereb Cortex. 2018;28(5):1685-1699. doi:10.1093/cercor/bhx066 28334314 PMC5907352

[30] Brown CA, Johnson NF, Anderson-Mooney AJ, . Development, validation and application of a new fornix template for studies of aging and preclinical Alzheimer’s disease. Neuroimage Clin. 2016;13:106-115. doi:10.1016/j.nicl.2016.11.024 27942453 PMC5137184

[31] Mori S, Oishi K, Jiang H, . Stereotaxic white matter atlas based on diffusion tensor imaging in an ICBM template. Neuroimage. 2008;40(2):570-582. doi:10.1016/j.neuroimage.2007.12.035 18255316 PMC2478641

[32] Archer D. Tractography templates for white matter microstructure analysis in aging and Alzheimer’s disease. Published online September 30, 2024.

[33] Davatzikos C, Xu F, An Y, Fan Y, Resnick SM. Longitudinal progression of Alzheimer’s-like patterns of atrophy in normal older adults: the SPARE-AD index. Brain. 2009;132(pt 8):2026-2035. doi:10.1093/brain/awp091 19416949 PMC2714059

[34] Landau SM, Ward TJ, Murphy A, ; Alzheimer’s Disease Neuroimaging Initiative. Quantification of amyloid beta and tau PET without a structural MRI. Alzheimers Dement. 2023;19(2):444-455. doi:10.1002/alz.12668 35429219

[35] Pascoal TA, Benedet AL, Tudorascu DL, . Longitudinal 18F-MK-6240 tau tangles accumulation follows Braak stages. Brain. 2021;144(11):3517-3528. doi:10.1093/brain/awab248 34515754 PMC8677534

[36] Pascoal TA, Therriault J, Benedet AL, . 18F-MK-6240 PET for early and late detection of neurofibrillary tangles. Brain. 2020;143(9):2818-2830. doi:10.1093/brain/awaa180 32671408

[37] Younes K, Smith V, Johns E, ; Alzheimer’s Disease Neuroimaging Initiative Researchers. Temporal tau asymmetry spectrum influences divergent behavior and language patterns in Alzheimer’s disease. Brain Behav Immun. 2024;119:807-817. doi:10.1016/j.bbi.2024.05.002 38710339 PMC12188992

[38] Duering M, Finsterwalder S, Baykara E, . Free water determines diffusion alterations and clinical status in cerebral small vessel disease. Alzheimers Dement. 2018;14(6):764-774. doi:10.1016/j.jalz.2017.12.007 29406155 PMC5994358

[39] Dumont M, Roy M, Jodoin PM, ; Alzheimer’s Disease Neuroimaging Initiative. Free Water in white matter differentiates MCI and AD from control subjects. Front Aging Neurosci. 2019;11:270. doi:10.3389/fnagi.2019.00270 31632265 PMC6783505

[40] Schouten TM, Koini M, Vos F, . Individual classification of Alzheimer’s disease with diffusion magnetic resonance imaging. Neuroimage. 2017;152:476-481. doi:10.1016/j.neuroimage.2017.03.025 28315741

[41] Dalboni da Rocha JL, Bramati I, Coutinho G, Tovar Moll F, Sitaram R. Fractional anisotropy changes in parahippocampal cingulum due to Alzheimer’s disease. Sci Rep. 2020;10(1):2660. doi:10.1038/s41598-020-59327-2 32060334 PMC7021702

[42] Nir TM, Jahanshad N, Villalon-Reina JE, ; Alzheimer’s Disease Neuroimaging Initiative (ADNI). Effectiveness of regional DTI measures in distinguishing Alzheimer’s disease, MCI, and normal aging. Neuroimage Clin. 2013;3:180-195. doi:10.1016/j.nicl.2013.07.006 24179862 PMC3792746

[43] Bozzali M, Giulietti G, Basile B, . Damage to the cingulum contributes to Alzheimer’s disease pathophysiology by deafferentation mechanism. Hum Brain Mapp. 2012;33(6):1295-1308. doi:10.1002/hbm.21287 21520352 PMC6870125

[44] Sexton CE, Kalu UG, Filippini N, Mackay CE, Ebmeier KP. A meta-analysis of diffusion tensor imaging in mild cognitive impairment and Alzheimer’s disease. Neurobiol Aging. 2011;32(12):2322.e5-2322.e18. doi:10.1016/j.neurobiolaging.2010.05.019 20619504

[45] Friedman NP, Robbins TW. The role of prefrontal cortex in cognitive control and executive function. Neuropsychopharmacology. 2022;47(1):72-89. doi:10.1038/s41386-021-01132-0 34408280 PMC8617292

[46] Miller EK, Cohen JD. An integrative theory of prefrontal cortex function. Annu Rev Neurosci. 2001;24:167-202. doi:10.1146/annurev.neuro.24.1.167 11283309

[47] Rafiq M, Jucla M, Guerrier L, Péran P, Pariente J, Pistono A. The functional connectivity of language network across the life span: Disentangling the effects of typical aging from Alzheimer’s disease. Front Aging Neurosci. 2022;14:959405. doi:10.3389/fnagi.2022.959405 36212038 PMC9537133

[48] Hertrich I, Dietrich S, Ackermann H. The role of the supplementary motor area for speech and language processing. Neurosci Biobehav Rev. 2016;68:602-610. doi:10.1016/j.neubiorev.2016.06.030 27343998

[49] Archer DB, Moore EE, Pamidimukkala U, . The relationship between white matter microstructure and self-perceived cognitive decline. Neuroimage Clin. 2021;32:102794. doi:10.1016/j.nicl.2021.102794 34479171 PMC8414539

[50] Ofori E, DeKosky ST, Febo M, ; Alzheimer’s Disease Neuroimaging Initiative. Free-water imaging of the hippocampus is a sensitive marker of Alzheimer’s disease. Neuroimage Clin. 2019;24:101985. doi:10.1016/j.nicl.2019.101985 31470214 PMC6722298

[51] Mortamais M, Ash JA, Harrison J, . Detecting cognitive changes in preclinical Alzheimer’s disease: a review of its feasibility. Alzheimers Dement. 2017;13(4):468-492. doi:10.1016/j.jalz.2016.06.2365 27702618

