# Supplementary material for: White Matter Abnormalities and Cognition in Aging and Alzheimer Disease
Source: JAMA Neurol. 2025 Jun 9;82(8):825–36. doi: 10.1001/jamaneurol.2025.1601 (PMC12150229; doi:10.1001/jamaneurol.2025.1601)
Supplement: Supplement 1. — eMethods. Supplemental Methods eReferences eTable 1. Batch Groupings in Longitudinal ComBat of Our Main Free-Water Database eTable 2. Participants in Gray Matter x White Matter Interaction Analysis eTable 3. Participants in Amyloid Status x White Matter Interaction Analysis eTable 4. Participants in Tau Status x White Matter Interaction Analysis eFigure 1. White Matter Associations With Cognitive Performance eFigure 2. White Matter Associations With Cognitive Decline eFigure 3. White Matter Interactions With APOE-ε4 on Memory Performance eFigure 4. White Matter Interactions With Gray Matter Atrophy on Memory Performance eFigure 5. White Matter Interactions With PET-Derived Amyloid Positivity on Executive Function Performance [file jamaneurol-e251601-s001.pdf]

Supplemental Online Content

Peter C, Sathe A, Shashikumar N, et al; Alzheimer's Disease Sequencing Project Phenotype Harmonization Consortium (ADSP-PHC) Analyst Team; Alzheimer's Disease Neuroimaging Initiative (ADNI); Biomarkers of Cognitive Decline Among Normal Adults (BIOCARD) Study Team; Alzheimer's Disease Sequencing Project (ADSP). White matter abnormalities and cognition in aging and Alzheimer disease. *JAMA Neurol*. Published online June 9, 2025. doi:10.1001/jamaneurol.2025.1601

eMethods. Supplemental Methods

eReferences

eTable 1. Batch Groupings in *Longitudinal ComBat* of Our Main Free-Water Database

eTable 2. Participants in Gray Matter x White Matter Interaction Analysis

eTable 3. Participants in Amyloid Status x White Matter Interaction Analysis

eTable 4. Participants in Tau Status x White Matter Interaction Analysis

eFigure 1. White Matter Associations With Cognitive Performance

eFigure 2. White Matter Associations With Cognitive Decline

eFigure 3. White Matter Interactions With *APOE*-ε4 on Memory Performance

eFigure 4. White Matter Interactions With Gray Matter Atrophy on Memory Performance

eFigure 5. White Matter Interactions With PET-Derived Amyloid Positivity on Executive Function Performance

This supplemental material has been provided by the authors to give readers additional information about their work.

© 2025 Peter C et al. *JAMA Neurology*.

eMethods. Supplemental Methods

Cohort Details

ADNI (www.adni.loni.usc.edu) is an ongoing, longitudinal, multicenter study designed to develop clinical, imaging, genetic, and biochemical biomarkers for the early detection and tracking of AD20. The current study used data from three ADNI phases, including ADNI-GO, ADNI 2, and ADNI 3. BLSA is a prospective cohort study with continuous enrollment that began in 1958[1]. BLSA data in the present study were collected between January 2009 and December 2018. Comprehensive data from BLSA are available upon request by a proposal submission through the cohort website (www.blsa.nih.gov). The BIOCARD study is designed to identify biomarkers associated with progression from normal cognitive status to cognitive impairment or dementia, with a particular focus on Alzheimer's Disease[2]. NACC maintains a centralized data repository for the National Institute of Aging's (NIA's) Alzheimer's Disease Research Centers (ADRC) Program, which currently includes 33 centers and 4 exploratory centers across the United States[3]. ROS/MAP/MARS are longitudinal, epidemiological clinical-pathological cohort studies that were designed to characterize common chronic conditions of aging and the neuropathological basis of cognitive impairment. ROS began in 1994 and enrolls older religious clergy from across the United States[4]. MAP was established in 1997 and enrolls lay persons from across northeastern Illinois[4]. MARS started in 2004 and enrolls older adults without dementia who self-identify as Black[5]. Notably, the three cohorts are managed by a single team with a large common core and neuroimaging data processed through a single pipeline, enabling efficient integration of data[6]. VMAP began in 2012 with the goal of investigating vascular health and brain aging[7]. WRAP was established in 2004 and is a study of midlife adults enriched for persons with a parental history of probable AD[8].

eReferences

[1] Shock NW, Greulich RC, Costa PT, et al. Normal Human Aging: The Baltimore Longitudinal Study on Aging. Published online 1984. Accessed June 26, 2024. http://hdl.handle.net/1903/23229
[2] Albert M, Soldan A, Gottesman R, et al. Cognitive changes preceding clinical symptom onset of mild cognitive impairment and relationship to ApoE genotype. Curr Alzheimer Res. 2014;11(8):773-784. doi:10.2174/1567205011081409101219200
[3] Weintraub S, Besser L, Dodge HH, et al. Version 3 of the Alzheimer Disease Centers' Neuropsychological Test Battery in the Uniform Data Set (UDS). Alzheimer Dis Assoc Disord. 2018;32(1):10-17. doi:10.1097/WAD.0000000000000223
[4] Bennett DA, Buchman AS, Boyle PA, Barnes LL, Wilson RS, Schneider JA. Religious Orders Study and Rush Memory and Aging Project. J Alzheimers Dis JAD. 2018;64(s1):S161-S189. doi:10.3233/JAD-179939
[5] Barnes LL, Shah RC, Aggarwal NT, Bennett DA, Schneider JA. The Minority Aging Research Study: ongoing efforts to obtain brain donation in African Americans without dementia. Curr Alzheimer Res. 2012;9(6):734-745. doi:10.2174/156720512801322627
[6] Makkinejad N, Evia AM, Tamhane AA, et al. ARTS: A novel In-vivo classifier of arteriolosclerosis for the older adult brain. NeuroImage Clin. 2021;31:102768. doi:10.1016/j.nicl.2021.102768

© 2025 Peter C et al. *JAMA Neurology*.

[7] Moore EE, Liu D, Pechman KR, et al. Increased Left Ventricular Mass Index Is Associated With Compromised White Matter Microstructure Among Older Adults. J Am Heart Assoc. 2018;7(13). doi:10.1161/JAHA.118.009041
[8] Johnson SC, Koscik RL, Jonaitis EM, et al. The Wisconsin Registry for Alzheimer's Prevention: A review of findings and current directions. Alzheimers Dement Amst Neth. 2018;10:130-142. doi:10.1016/j.dadm.2017.11.007

© 2025 Peter C et al. *JAMA Neurology*.

eTable 1. Batch Groupings in *Longitudinal ComBat* of Our Main Free-Water Database

Batch	Original Cohort	Scanner Name	ADNI Phase	ADNI Shells	BLSA Scanner Num	NACCADC	RMM Site	VMAP Phase	Coil/Software Version
1	ADNI	GE Medical Systems	ADNI2	Singleshell	-	-	-	-	-
2	ADNI	GE Medical Systems	ADNI3	Singleshell	-	-	-	-	-
3	ADNI	GE Medical Systems	ADNIGO	Singleshell	-	-	-	-	-
4	ADNI	Philips Healthcare	ADNI3	Singleshell	-	-	-	-	-
5	ADNI	Philips Medical	ADNI3	Singleshell	-	-	-	-	-
6	ADNI	Philips	ADNI3	Singleshell	-	-	-	-	-
7	ADNI	Siemens Healtineers	ADNI3	Multishell	-	-	-	-	-
8	ADNI	Siemens	ADNI3	Singleshell	-	-	-	-	-
9	ADNI	Siemens	ADNI3	Multishell	-	-	-	-	-
10	BLSA	-	-	-	10	-	-	-	-
11	BLSA	-	-	-	7	-	-	-	-
12	BLSA	-	-	-	9	-	-	-	-
13	BIOCARD	-	-	-	-	-	-	-	-
14	NACC	Discovery MR750	-	-	-	2578	-	-	-
15	NACC	Discovery MR750	-	-	-	6499	-	-	-
16	NACC	Genesis Signa	-	-	-	6518	-	-	-
17	NACC	TrioTim	-	-	-	354	-	-	-
18	NACC	TrioTim	-	-	-	5783	-	-	-
19	NACC	TrioTim	-	-	-	6518	-	-	-
20	NACC	Verio	-	-	-	354	-	-	-
21	NACC	Discovery MR750	-	-	-	4032	-	-	-
22	NACC	Discovery MR750	-	-	-	8361	-	-	-
23	ROSMAPMARS	-	-	-	-	-	mg1	-	-
24	ROSMAPMARS	-	-	-	-	-	mg2	-	-
25	ROSMAPMARS	-	-	-	-	-	uc1	-	-
26	VMAP	3Ta	-	-	-	-	-	1	8chSENSE; 5.1.7.1
27	VMAP	3Tb	-	-	-	-	-	1	8chSENSE; 3.2.2.0
28	VMAP	3Tb	-	-	-	-	-	1	8chSENSE; 5.1.7.1
29	VMAP	3Tb	-	-	-	-	-	1	32chdStream; 5.3.0.3
30	VMAP	3Tb	-	-	-	-	-	1	8chdStream; 5.3.0.2
31	VMAP	3Tb	-	-	-	-	-	1	8chdStream; 5.3.0.3
32	VMAP	3Tb	-	-	-	-	-	2	32chdStream; 5.6.1.0
33	VMAP	3Tb	-	-	-	-	-	2	32chdStream; 5.9.0
34	WRAP	GE Medical Systems	-	-	-	-	-	-	-

© 2025 Peter C et al. *JAMA Neurology*.

eTable 2. Participants in Gray Matter x White Matter Interaction Analysis

Characteristic	Cohort				
	ADNI	NACC	ROS/MAP/MARS	WRAP	Total
Number of participants	829	824	1,051	271	2,975
Number of sessions	1,780	935	2,296	373	5,384
Average number of visits	2.15 (1.50)	1.13 (0.38)	2.18 (1.38)	1.38 (0.64)	1.81 (1.26)
Follow-up time, y	2.66 (2.10)	1.99 (1.05)	4.28 (2.51)	3.35 (1.61)	3.49 (2.41)
Age at baseline, y	74.75 (7.50)	74.78 (8.27)	79.62 (7.23)	62.44 (6.34)	75.36 (8.86)
Female sex, No. (%)	413 (49.82)	471 (57.16)	814 (77.45)	175 (64.58)	1,873 (62.96)
Education, y	16.33 (2.59)	14.74 (3.85)	15.89 (3.26)	16.67 (2.77)	15.77 (3.30)
NHW race, No. (%)	706 (85.16)	597 (72.45)	781 (74.31)	265 (97.79)	2,349 (78.96)
APOE-ε4 carrier, No. (%)	295 (35.59)	329 (39.93)	203 (19.31)	83 (30.63)	910 (30.59)
Baseline clinical status (CU; MCI; AD), No. (%)	393 (47.41); 324 (39.08); 112 (13.51)	467 (56.67); 227 (27.55); 130 (15.78)	844 (80.30); 193 (18.36); 14 (1.33)	268 (98.89); 2 (0.74); 1 (0.37)	1,972 (66.29); 746 (25.08); 257 (8.64)
Diagnosis converter, No. (%)	467 (56.33)	375 (45.51)	301 (28.64)	4 (1.48)	1,147 (38.55)
Left hippocampal volume at baseline (mm^3)	3,372 (470)	3,295 (445)	3,279 (388)	3,633 (381)	3,342 (439)
Right hippocampal volume at baseline (mm^3)	3,692 (490)	3,589 (477)	3,572 (425)	3,941 (421)	3,644 (470)
Intracranial volume at baseline (cm^3)	1,421 (143)	1,367 (143)	1,343 (136)	1,374 (123)	1,374 (142)
SPARE-AD	-0.12 (0.67)	-0.17 (0.64)	-0.18 (0.52)	-0.62 (0.40)	-0.20 (0.61)
Composite memory composite at baseline (z-score)	0.49 (0.86)	0.25 (0.89)	0.38 (0.54)	1.21 (0.42)	0.45 (0.78)
Composite executive function composite at baseline (z-score)	0.45 (0.67)	0.12 (0.80)	0.54 (0.55)	0.87 (0.31)	0.43 (0.68)
Composite language composite at baseline (z-score)	0.49 (0.64)	0.42 (0.76)	0.42 (0.58)	1.14 (0.35)	0.50 (0.67)

Abbreviations: ADNI, Alzheimer's Disease Neuroimaging Initiative; NACC, National Alzheimer's Coordinating Center; ROS, Religious Orders Study; MAP, Rush Memory and Aging Project; MARS, Minority Aging Research Study; WRAP, Wisconsin Registry for Alzheimer's Prevention; *APOE*-ε4, apolipoprotein ε4; CU, cognitively unimpaired; MCI, mild cognitive impairment; AD, Alzheimer's disease.

© 2025 Peter C et al. *JAMA Neurology*.

eTable 3. Participants in Amyloid Status x White Matter Interaction Analysis

Characteristic	Cohort			
	ADNI	NACC	WRAP	Total
Number of participants	538	31	124	693
Age at baseline, y	75.92 (7.11)	75.11 (6.72)	62.76 (6.36)	73.53 (8.59)
Female sex, No. (%)	270 (50.19)	18 (58.06)	86 (69.35)	374 (53.97)
Education, y	16.39 (2.57)	16.55 (2.50)	16.73 (2.92)	16.46 (2.64)
NHW race, No. (%)	479 (89.03)	30 (96.77)	121 (97.58)	630 (90.91)
APOE-ε4 carrier, No. (%)	207 (34.48)	10 (32.26)	36 (29.03)	253 (36.51)
Baseline clinical status (CU; MCI; AD), No. (%)	295 (54.83); 170 (31.60); 73 (13.57)	22 (70.97); 7 (22.58); 2 (6.45)	122 (98.39); 1 (0.81); 1 (0.81)	439 (63.35); 178 (25.69); 76 (10.97)
Diagnosis converter, No. (%)	266 (49.44)	9 (29.03)	2 (1.61)	277 (39.97)
Amyloid Positive, No. (%)	226 (42.01)	12 (38.71)	18 (15.52)	256 (36.94)
Composite memory composite at baseline (z-score)	0.56 (0.90)	0.78 (1.06)	1.21 (0.41)	0.69 (0.87)
Composite executive function composite at baseline (z-score)	0.51 (0.69)	0.40 (0.72)	0.86 (0.30)	0.57 (0.65)
Composite language composite at baseline (z-score)	0.56 (0.67)	0.54 (0.86)	1.11 (0.33)	0.66 (0.66)

Abbreviations: ADNI, Alzheimer's Disease Neuroimaging Initiative; NACC, National Alzheimer's Coordinating Center; WRAP, Wisconsin Registry for Alzheimer's Prevention; *APOE*-ε4, apolipoprotein ε4; CU, cognitively unimpaired; MCI, mild cognitive impairment; AD, Alzheimer's disease.

© 2025 Peter C et al. *JAMA Neurology*.

eTable 4. Participants in Tau Status x White Matter Interaction Analysis

Characteristic	Cohort			
	ADNI	NACC	WRAP	Total
Number of participants	397	21	16	434
Age at baseline, y	75.84 (7.08)	74.85 (6.01)	68.55 (5.52)	75.53 (7.11)
Female sex, No. (%)	201 (50.63)	15 (71.43)	9 (56.25)	225 (51.84)
Education, y	16.41 (2.57)	16.43 (2.48)	15.88 (2.99)	16.39 (2.58)
NHW race, No. (%)	346 (87.15)	20 (95.24)	16 (100)	382 (88.02)
APOE-ε4 carrier, No. (%)	148 (37.28)	3 (14.29)	6 (37.50)	157 (36.18)
Baseline clinical status (CU; MCI; AD), No. (%)	213 (53.65); 129 (32.49); 55 (13.95)	20 (95.24); 1 (4.76); 0 (0)	15 (93.75); 1 (6.25); 0 (0)	248 (57.14); 130 (29.95); 56 (12.90)
Diagnosis converter, No. (%)	201 (50.63)	1 (4.76)	1 (6.25)	203 (46.77)
Tau Positive, No. (%)	136 (34.26)	4 (19.05)	3 (18.75)	143 (32.95)
Composite memory composite at baseline (z-score)	0.53 (0.87)	1.09 (1.08)	1.38 (0.62)	0.59 (0.90)
Composite executive function composite at baseline (z-score)	0.52 (0.71)	0.39 (0.75)	0.92 (0.32)	0.53 (0.70)
Composite language composite at baseline (z-score)	0.57 (0.65)	0.63 (0.84)	1.19 (0.43)	0.59 (0.66)

Abbreviations: ADNI, Alzheimer's Disease Neuroimaging Initiative; NACC, National Alzheimer's Coordinating Center; WRAP, Wisconsin Registry for Alzheimer's Prevention; *APOE*-ε4, apolipoprotein ε4; CU, cognitively unimpaired; MCI, mild cognitive impairment; AD, Alzheimer's disease.

© 2025 Peter C et al. *JAMA Neurology.*

eFigure 1. White Matter Associations With Cognitive Performance

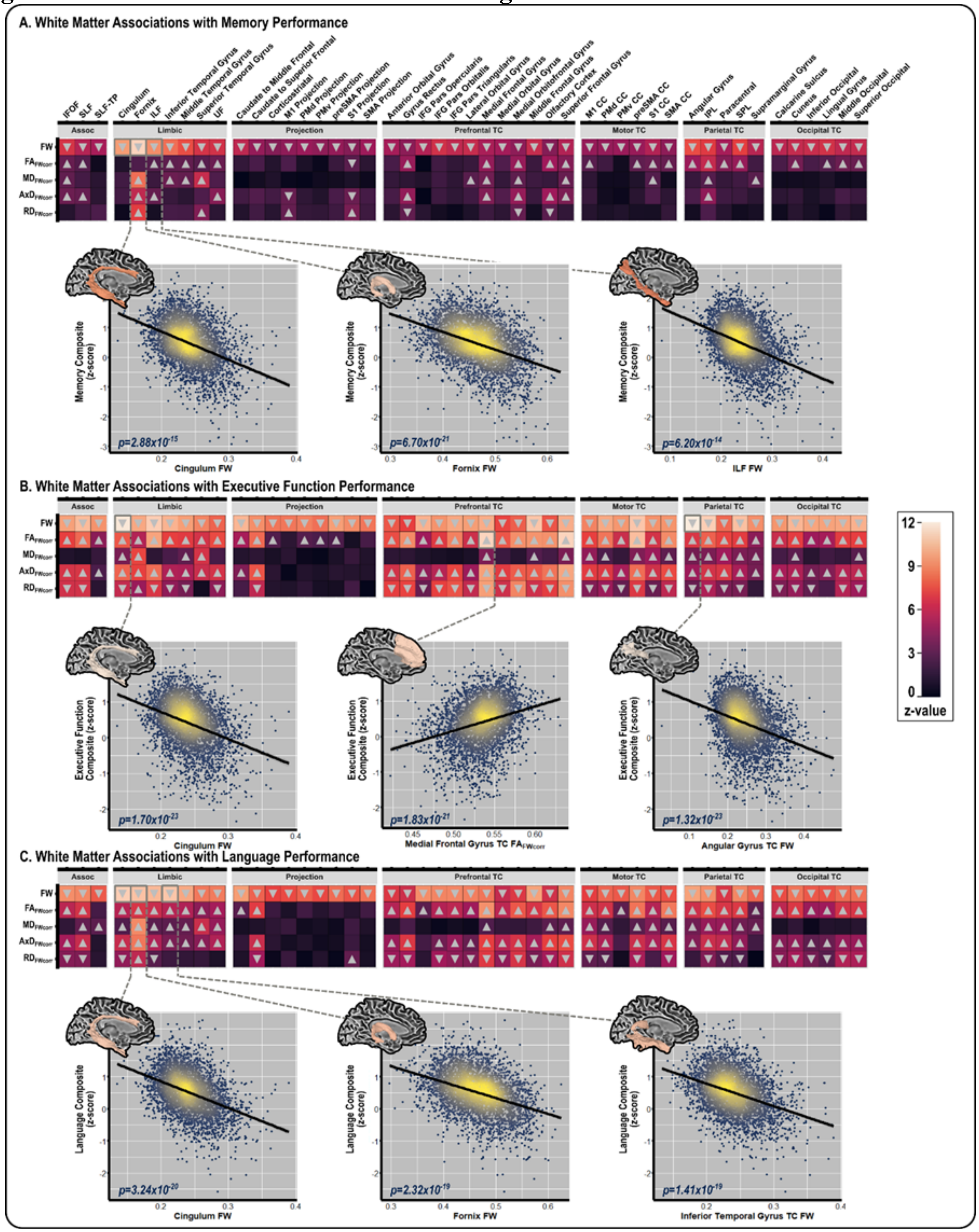

The association between FW-corrected metrics and baseline cognitive performance across the memory (**A**), executive function (**B**), and language (**C**) domains. Linear regression models were conducted for each FW-corrected metric (FW, FA_{FWcorr}, MD_{FWcorr}, AxD_{FWcorr}, RD_{FWcorr}). Each heatmap is grouped by tract-type and represents the individual z-value for each independent model. The arrows represent the direction of beta coefficients which reached significance following correction for multiple comparisons. The regression plots show the correlations between cognitive performance and the top three most sensitive microstructural measures for each domain.

© 2025 Peter C et al. *JAMA Neurology.*

eFigure 2. White Matter Associations With Cognitive Decline

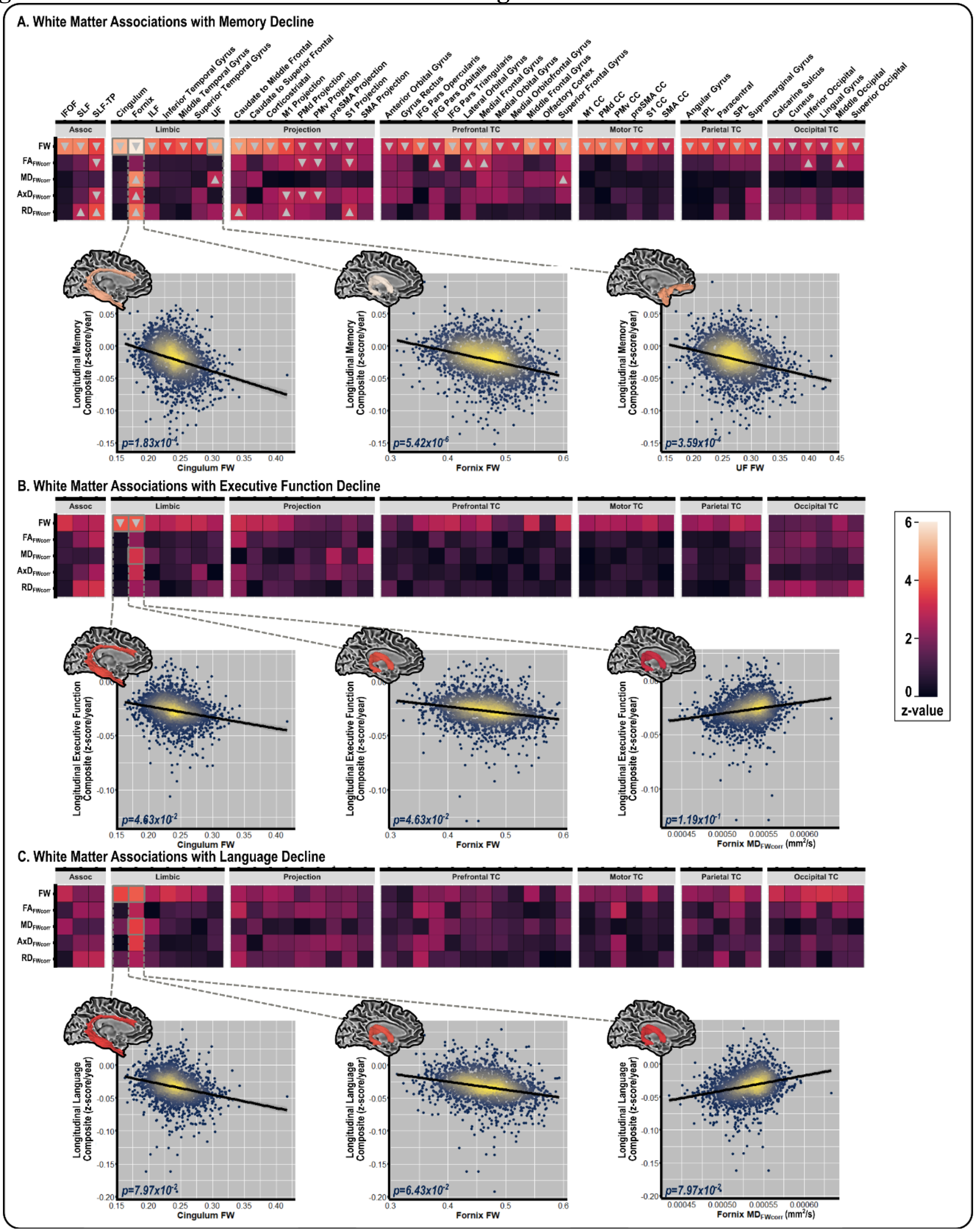

The association between baseline FW-corrected metrics and longitudinal cognitive decline across the memory (**A**), executive function (**B**), and language (**C**) domains. Linear mixed-effect regression models were conducted for each FW-corrected metric (FW, FA_{FWcorr}, MD_{FWcorr}, AxD_{FWcorr}, RD_{FWcorr}). Each heatmap is grouped by tract-type and represents the individual z-value for each independent model. The arrows represent the direction of beta coefficients which reached significance following correction for multiple comparisons. The regression plots show the correlations between cognitive performance and the top three most sensitive microstructural measures for each domain.

© 2025 Peter C et al. *JAMA Neurology.*

eFigure 3. White Matter Interactions With *APOE*-ε4 on Memory Performance

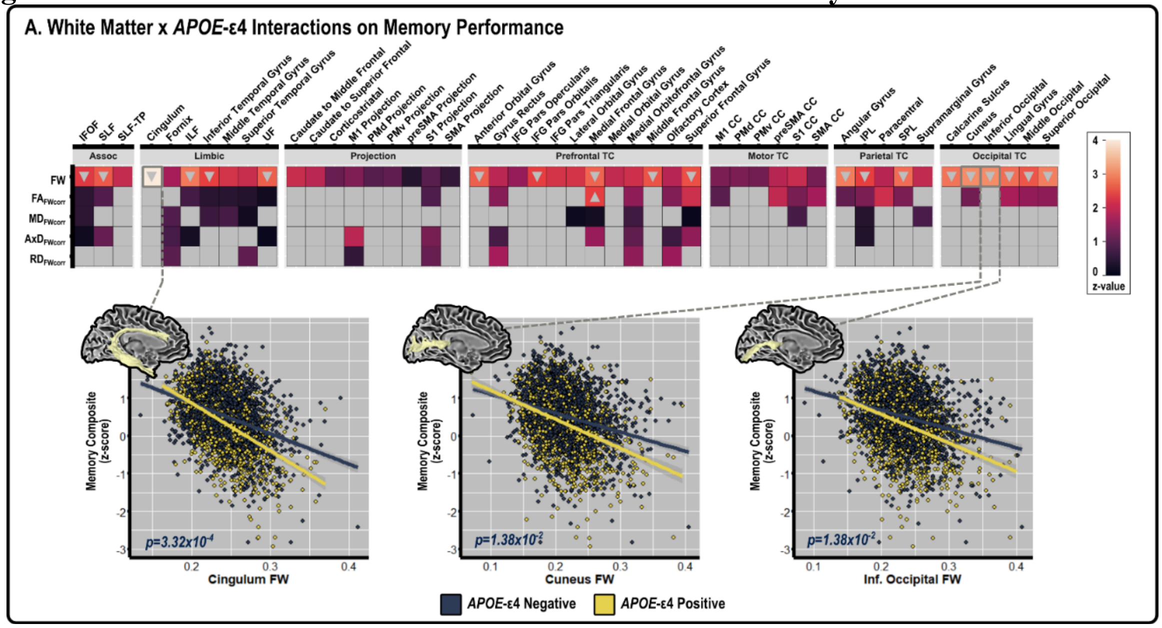

This figure illustrates the interaction of FW-corrected metrics and *APOE*-ε4 positivity on baseline memory performance. Interaction models were conducted for each FW-corrected metric (FW, FA_{FWcorr}, MD_{FWcorr}, AxD_{FWcorr}, RD_{FWcorr}). The heatmap, grouped by tract type, displays the individual interaction test statistic for each independent model. Arrows indicate the direction of significant beta coefficients: downward arrows signify that *APOE*-ε4 positivity interacted with higher white matter microstructural abnormalities to predict poorer memory performance. Regression plots highlight the interactions on cognitive performance for the top three most sensitive microstructural measures, stratified by *APOE*-ε4 status (blue=*APOE*-ε4 negative; yellow=*APOE*-ε4 positive).

© 2025 Peter C et al. *JAMA Neurology*.

eFigure 4. White Matter Interactions With Gray Matter Atrophy on Memory Performance

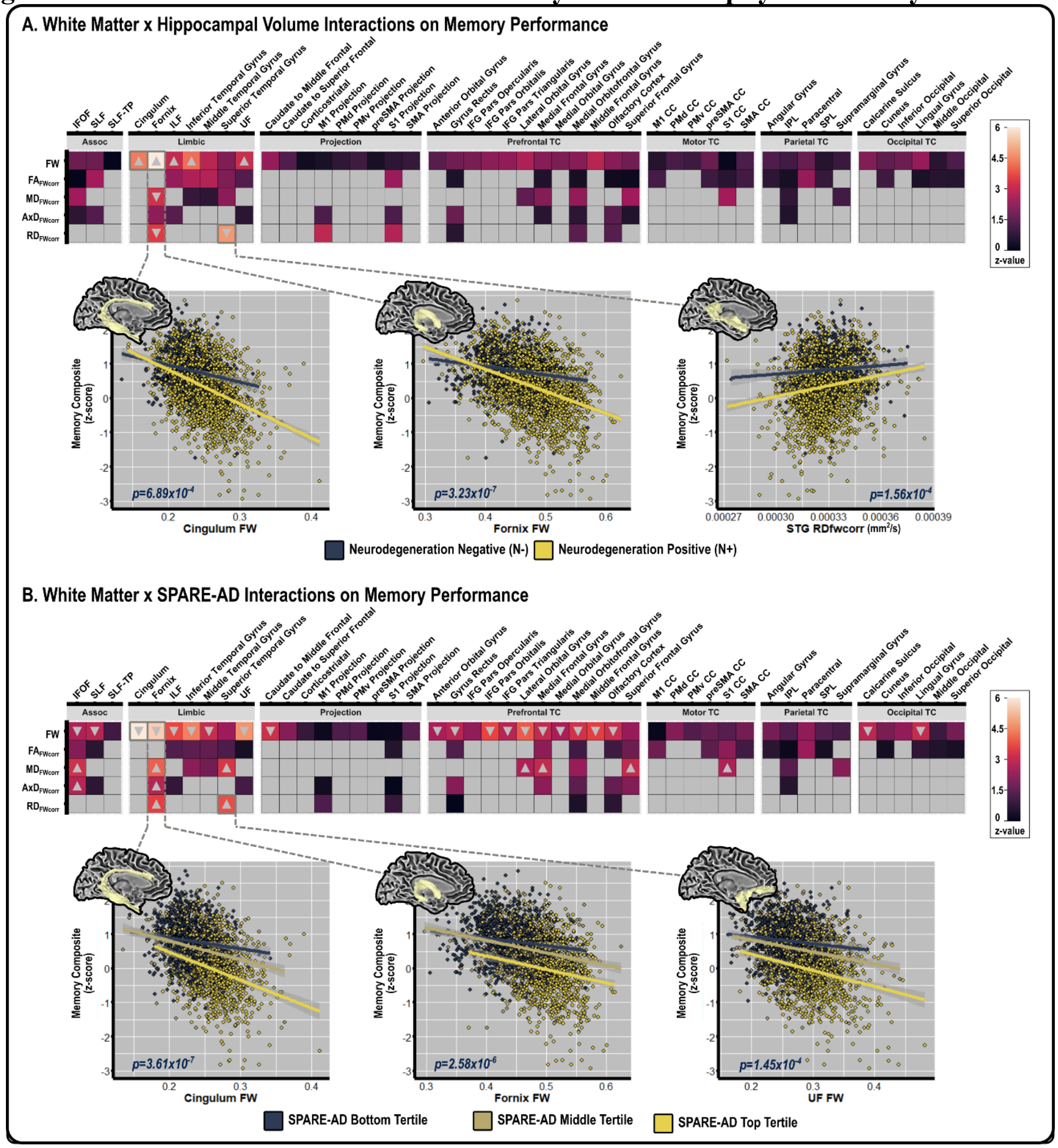

This figure illustrates the interactions between hippocampal volume (**A**) and the SPARE-AD index (**B**) with FW-corrected metrics on baseline memory performance (n=2,975). Interaction models were conducted for each FW-corrected metric (FW, FA_{FWcorr}, MD_{FWcorr}, AxD_{FWcorr}, RD_{FWcorr}). The heatmaps, grouped by tract type, display the individual interaction test statistic for each model. Regression plots highlight the interactions on memory performance for the top three most sensitive microstructural measures, stratified by hippocampal atrophy status (blue = neurodegenerative negative [volume>6,723 mm³]; yellow = neurodegenerative positive [volume≤6,723 mm³]) or SPARE-AD index tertile (blue = bottom tertile; gold = middle tertile; yellow = top tertile).

© 2025 Peter C et al. *JAMA Neurology.*

eFigure 5. White Matter Interactions With PET-Derived Amyloid Positivity on Executive Function Performance

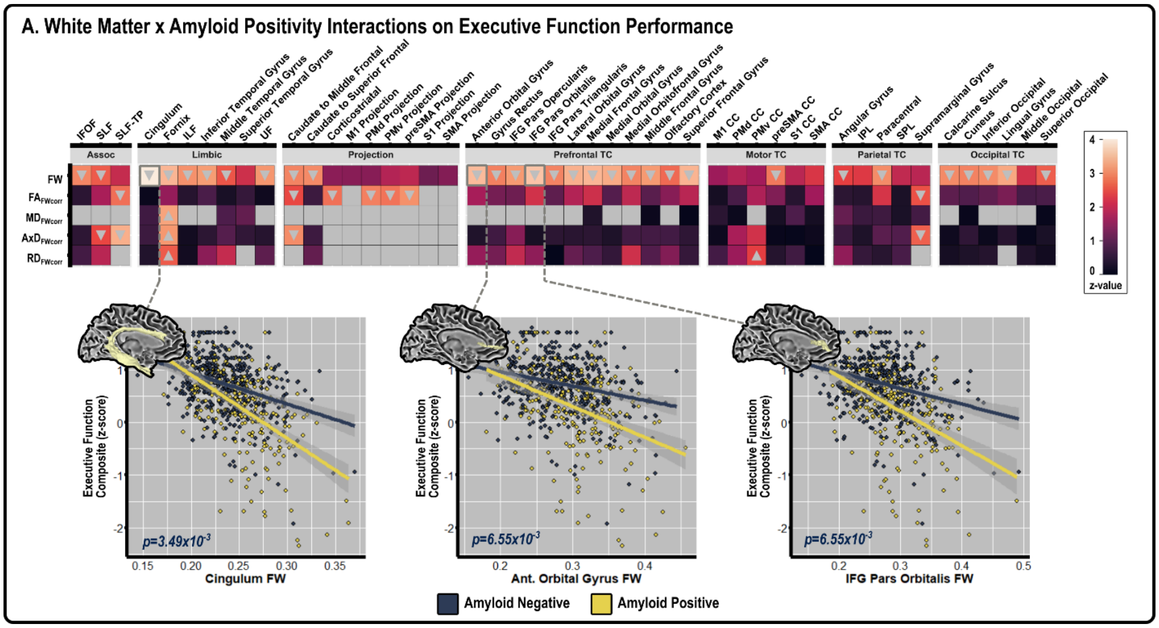

This figure illustrates the interaction of FW-corrected metrics and amyloid PET positivity on baseline executive function performance (n=693). Interaction models were conducted for each FW-corrected metric (FW, FA_{FWcorr}, MD_{FWcorr}, AxD_{FWcorr}, RD_{FWcorr}). The heatmap, grouped by tract type, displays the individual interaction test statistic for each independent model. Arrows indicate the direction of significant beta coefficients: downward arrows signify that amyloid positivity interacted with higher white matter microstructural abnormalities to predict poorer memory performance. Regression plots highlight the interactions on cognitive performance for the top three most sensitive microstructural measures, stratified by amyloid status (blue=amyloid negative; yellow=amyloid positive).

© 2025 Peter C et al. *JAMA Neurology.*
