# Supplementary material for: White Matter Abnormalities and Cognition in Aging and Alzheimer Disease
Source: JAMA Neurol. 2025 Jun 9;82(8):825–36. doi: 10.1001/jamaneurol.2025.1601 (PMC12150229; doi:10.1001/jamaneurol.2025.1601)
Supplement: Supplement 3. — Members of the Alzheimer’s Disease Neuroimaging Initiative (ADNI), Biomarkers of Cognitive Decline Among Normal Adults (BIOCARD) Study Team, Alzheimer's Disease Sequencing Project (ADSP) [file jamaneurol-e251601-s003.pdf]

Supplemental Online Content: Nonauthor Collaborators
*First name, last name, and suffix (if applicable) are required and will appear in PubMed.

***Group Name(s): Alzheimer's Disease Neuroimaging Initiative (ADNI), Biomarkers of Cognitive Decline Among Normal Adults (BIOCARD) Study Team, Alzheimer's Disease Sequencing Project (ADSP)**

*First Name and Middle Initial(s)	*Last Name	*Suffix (eg, Jr, III)	Academic Degrees	Institution	Location (city, state/province, country)	Role or Contribution, eg, chair, principal investigator	Group (if more than 1 Group listed in the byline) and/or Subgroup (eg, Steering Committee)
Olusegun	Adegoke		MSc				Alzheimer's Disease Neuroimaging Initiative (ADNI)
Paul	Aisen		MD				Alzheimer's Disease Neuroimaging Initiative (ADNI)
Liana G.	Apostolova		MD				Alzheimer's Disease Neuroimaging Initiative (ADNI)
Miriam	Ashford		PhD				Alzheimer's Disease Neuroimaging Initiative (ADNI)
Lisa	Barnes		PhD				Alzheimer's Disease Neuroimaging Initiative (ADNI)
Laurel	Beckett		PhD				Alzheimer's Disease Neuroimaging Initiative (ADNI)
Marie	Bernard		MD				Alzheimer's Disease Neuroimaging Initiative (ADNI)
Haley	Bernhardt		BA, R. EEG T				Alzheimer's Disease Neuroimaging Initiative (ADNI)
Bret	Borowski		RTR				Alzheimer's Disease Neuroimaging Initiative (ADNI)
Yuliana	Cabrera		BS				Alzheimer's Disease Neuroimaging Initiative (ADNI)
Nigel J.	Cairns		PhD, FRCPath				Alzheimer's Disease Neuroimaging Initiative (ADNI)
Maria	Carrillo		PhD				Alzheimer's Disease Neuroimaging Initiative (ADNI)
Kewei	Chen		PhD				Alzheimer's Disease Neuroimaging Initiative (ADNI)
Mark	Choe		BS				Alzheimer's Disease Neuroimaging Initiative (ADNI)
Taylor	Clanton		MPH				Alzheimer's Disease Neuroimaging Initiative (ADNI)
Godfrey	Coker		MBA, MPH				Alzheimer's Disease Neuroimaging Initiative (ADNI)
Cat	Conti		BA				Alzheimer's Disease Neuroimaging Initiative (ADNI)
Karen	Crawford						Alzheimer's Disease Neuroimaging Initiative (ADNI)
Sandhitsu	Das		PhD				Alzheimer's Disease Neuroimaging Initiative (ADNI)
Charles	DeCarli		MD				Alzheimer's Disease Neuroimaging Initiative (ADNI)
Michael	Donohue		PhD				Alzheimer's Disease Neuroimaging Initiative (ADNI)
Kelley	Faber		MS, CCRC				Alzheimer's Disease Neuroimaging Initiative (ADNI)
Adam	Fleisher		MD				Alzheimer's Disease Neuroimaging Initiative (ADNI)
Derek	Flenneiken						Alzheimer's Disease Neuroimaging Initiative (ADNI)
Evan	Fletcher		PhD				Alzheimer's Disease Neuroimaging Initiative (ADNI)
Juliet	Fockler						Alzheimer's Disease Neuroimaging Initiative (ADNI)
Arvin	Forghanian-Arani		PhD				Alzheimer's Disease Neuroimaging Initiative (ADNI)
Tatiana M.	Foroud		PhD				Alzheimer's Disease Neuroimaging Initiative (ADNI)
Nick C.	Fox		MD				Alzheimer's Disease Neuroimaging Initiative (ADNI)
Erin	Franklin		MS				Alzheimer's Disease Neuroimaging Initiative (ADNI)
Devon	Gessert		BS				Alzheimer's Disease Neuroimaging Initiative (ADNI)
Hector	González						Alzheimer's Disease Neuroimaging Initiative (ADNI)
Robert C.	Green		MD, MPH				Alzheimer's Disease Neuroimaging Initiative (ADNI)
Jeffery	Gunter		PhD				Alzheimer's Disease Neuroimaging Initiative (ADNI)
Danielle	Harvey		PhD				Alzheimer's Disease Neuroimaging Initiative (ADNI)
Lindsey	Hergesheimer		BS				Alzheimer's Disease Neuroimaging Initiative (ADNI)
Carole	Ho						Alzheimer's Disease Neuroimaging Initiative (ADNI)
Erin	Householder		MS				Alzheimer's Disease Neuroimaging Initiative (ADNI)
John K.	Hsaio		MD				Alzheimer's Disease Neuroimaging Initiative (ADNI)
Clifford R.	Jack	Jr	MD				Alzheimer's Disease Neuroimaging Initiative (ADNI)
Jonathan	Jackson		PhD				Alzheimer's Disease Neuroimaging Initiative (ADNI)
William	Jagust		MD				Alzheimer's Disease Neuroimaging Initiative (ADNI)
Neda	Jahanshad		PhD				Alzheimer's Disease Neuroimaging Initiative (ADNI)
Gustavo	Jimenez		MBS				Alzheimer's Disease Neuroimaging Initiative (ADNI)

Supplemental Online Content: Nonauthor Collaborators
*First name, last name, and suffix (if applicable) are required and will appear in PubMed.

*First Name and Middle Initial(s)	*Last Name	*Suffix (eg, Jr, III)	Academic Degrees	Institution	Location (city, state/province, country)	Role or Contribution, eg, chair, principal investigator	Group (if more than 1 Group listed in the byline) and/or Subgroup (eg, Steering Committee)
Chengshu	Jin		PhD				Alzheimer's Disease Neuroimaging Initiative (ADNI)
David	Jones		MD				Alzheimer's Disease Neuroimaging Initiative (ADNI)
Kejal	Kantarci		MD				Alzheimer's Disease Neuroimaging Initiative (ADNI)
Zaven	Khachaturian		PhD				Alzheimer's Disease Neuroimaging Initiative (ADNI)
Alexander	Knaack		MS				Alzheimer's Disease Neuroimaging Initiative (ADNI)
Robert A.	Koeppe		PhD				Alzheimer's Disease Neuroimaging Initiative (ADNI)
Adrienne	Kormos						Alzheimer's Disease Neuroimaging Initiative (ADNI)
Susan	Landau		PhD				Alzheimer's Disease Neuroimaging Initiative (ADNI)
Payam	Mahboubi		MPH				Alzheimer's Disease Neuroimaging Initiative (ADNI)
Ian	Malone		PhD				Alzheimer's Disease Neuroimaging Initiative (ADNI)
Eliezer	Masliah		MD				Alzheimer's Disease Neuroimaging Initiative (ADNI)
Donna	Masterman		MD				Alzheimer's Disease Neuroimaging Initiative (ADNI)
Chet	Mathis		MD				Alzheimer's Disease Neuroimaging Initiative (ADNI)
Garrett	Miller		MS				Alzheimer's Disease Neuroimaging Initiative (ADNI)
Tom	Montine		MD, PhD				Alzheimer's Disease Neuroimaging Initiative (ADNI)
Shelley	Moore		BA				Alzheimer's Disease Neuroimaging Initiative (ADNI)
John C.	Morris		MD				Alzheimer's Disease Neuroimaging Initiative (ADNI)
Scott	Neu		PhD				Alzheimer's Disease Neuroimaging Initiative (ADNI)
John	Neuhaus		PhD				Alzheimer's Disease Neuroimaging Initiative (ADNI)
Kwangsik	Nho		PhD				Alzheimer's Disease Neuroimaging Initiative (ADNI)
Talia M.	Nir		PhD				Alzheimer's Disease Neuroimaging Initiative (ADNI)
Rachel	Nosheny		PhD				Alzheimer's Disease Neuroimaging Initiative (ADNI)
Kelly	Nudelman		PhD				Alzheimer's Disease Neuroimaging Initiative (ADNI)
Ozioma	Okonkwo		PhD				Alzheimer's Disease Neuroimaging Initiative (ADNI)
Richard J.	Perrin		MD, PhD				Alzheimer's Disease Neuroimaging Initiative (ADNI)
Ronald	Peterson		MD, PhD				Alzheimer's Disease Neuroimaging Initiative (ADNI)
Jeremy	Pizzola		BA				Alzheimer's Disease Neuroimaging Initiative (ADNI)
William	Potter		MD				Alzheimer's Disease Neuroimaging Initiative (ADNI)
Michael	Rafii		MD, PhD				Alzheimer's Disease Neuroimaging Initiative (ADNI)
Rema	Raman		PhD				Alzheimer's Disease Neuroimaging Initiative (ADNI)
Robert	Reid		PhD				Alzheimer's Disease Neuroimaging Initiative (ADNI)
Eric R.	Reiman		MD				Alzheimer's Disease Neuroimaging Initiative (ADNI)
Shannon L.	Risacher		PhD				Alzheimer's Disease Neuroimaging Initiative (ADNI)
Stephanie	Rossi Chen		BA				Alzheimer's Disease Neuroimaging Initiative (ADNI)
Laurie	Ryan		PhD				Alzheimer's Disease Neuroimaging Initiative (ADNI)
Jennifer	Salazar		MBS				Alzheimer's Disease Neuroimaging Initiative (ADNI)
Andrew J.	Saykin		PsyD				Alzheimer's Disease Neuroimaging Initiative (ADNI)
Christopher	Schwarz		PhD				Alzheimer's Disease Neuroimaging Initiative (ADNI)
Matthew	Senjem		MS				Alzheimer's Disease Neuroimaging Initiative (ADNI)
Elizabeth	Shaffer		BS				Alzheimer's Disease Neuroimaging Initiative (ADNI)
Leslie M.	Shaw		PhD				Alzheimer's Disease Neuroimaging Initiative (ADNI)
Li	Shen		PhD				Alzheimer's Disease Neuroimaging Initiative (ADNI)
Nina	Silverberg		PhD				Alzheimer's Disease Neuroimaging Initiative (ADNI)
Stephanie	Smith		BS				Alzheimer's Disease Neuroimaging Initiative (ADNI)
Lisa	Taylor-Reinwald		BA, HTL				Alzheimer's Disease Neuroimaging Initiative (ADNI)
Leon	Thal		MD				Alzheimer's Disease Neuroimaging Initiative (ADNI)

*First name, last name, and suffix (if applicable) are required and will appear in PubMed.

*First Name and Middle Initial(s)	*Last Name	*Suffix (eg, Jr, III)	Academic Degrees	Institution	Location (city, state/province, country)	Role or Contribution, eg, chair, principal investigator	Group (if more than 1 Group listed in the byline) and/or Subgroup (eg, Steering Committee)
Sophia I.	Thomopoulos		BS				Alzheimer's Disease Neuroimaging Initiative (ADNI)
Paul	Thompson		PhD				Alzheimer's Disease Neuroimaging Initiative (ADNI)
Arthur W.	Toga		PhD				Alzheimer's Disease Neuroimaging Initiative (ADNI)
Duygu	Tosun-Turgut		PhD				Alzheimer's Disease Neuroimaging Initiative (ADNI)
John Q.	Trojanowski		MD, PhD				Alzheimer's Disease Neuroimaging Initiative (ADNI)
Diana	Truran Sacrey						Alzheimer's Disease Neuroimaging Initiative (ADNI)
Dallas	Veitch		PhD				Alzheimer's Disease Neuroimaging Initiative (ADNI)
Prashanthi	Vemuri		PhD				Alzheimer's Disease Neuroimaging Initiative (ADNI)
Sarah	Walter		MSc				Alzheimer's Disease Neuroimaging Initiative (ADNI)
Chad	Ward						Alzheimer's Disease Neuroimaging Initiative (ADNI)
Michael W.	Weiner		MD				Alzheimer's Disease Neuroimaging Initiative (ADNI)
Kristi	Wilmes		MS, CCRP				Alzheimer's Disease Neuroimaging Initiative (ADNI)
Paul A.	Yushkevich		PhD				Alzheimer's Disease Neuroimaging Initiative (ADNI)
Caileigh	Zimmerman		MS				Alzheimer's Disease Neuroimaging Initiative (ADNI)
Marilyn	Albert					Administrative Core	Biomarkers of Cognitive Decline Among Normal Adults (BIOCARD) Study Team
Barbara	Rodzon					Administrative Core	Biomarkers of Cognitive Decline Among Normal Adults (BIOCARD) Study Team
Marilyn	Albert					Clinical Core	Biomarkers of Cognitive Decline Among Normal Adults (BIOCARD) Study Team
Anja	Soldan					Clinical Core	Biomarkers of Cognitive Decline Among Normal Adults (BIOCARD) Study Team
Corinne	Pettigrew					Clinical Core	Biomarkers of Cognitive Decline Among Normal Adults (BIOCARD) Study Team
Leonie	Farrington					Clinical Core	Biomarkers of Cognitive Decline Among Normal Adults (BIOCARD) Study Team
Maura	Grega					Clinical Core	Biomarkers of Cognitive Decline Among Normal Adults (BIOCARD) Study Team
Gay	Rudow					Clinical Core	Biomarkers of Cognitive Decline Among Normal Adults (BIOCARD) Study Team
Scott	Rudow					Clinical Core	Biomarkers of Cognitive Decline Among Normal Adults (BIOCARD) Study Team
Michael	Miller					Imaging Core	Biomarkers of Cognitive Decline Among Normal Adults (BIOCARD) Study Team
Arnold	Bakker					Imaging Core	Biomarkers of Cognitive Decline Among Normal Adults (BIOCARD) Study Team
Tilak	Ratnanather					Imaging Core	Biomarkers of Cognitive Decline Among Normal Adults (BIOCARD) Study Team
Anthony	Kolasny					Imaging Core	Biomarkers of Cognitive Decline Among Normal Adults (BIOCARD) Study Team
Kenichi	Oishi					Imaging Core	Biomarkers of Cognitive Decline Among Normal Adults (BIOCARD) Study Team
Laurent	Younes					Imaging Core	Biomarkers of Cognitive Decline Among Normal Adults (BIOCARD) Study Team
Abhay	Moghekar					Biospecimen Core	Biomarkers of Cognitive Decline Among Normal Adults (BIOCARD) Study Team

Supplemental Online Content: Nonauthor Collaborators
*First name, last name, and suffix (if applicable) are required and will appear in PubMed.

*First Name and Middle Initial(s)	*Last Name	*Suffix (eg, Jr, III)	Academic Degrees	Institution	Location (city, state/province, country)	Role or Contribution, eg, chair, principal investigator	Group (if more than 1 Group listed in the byline) and/or Subgroup (eg, Steering Committee)
Chan-Hyun	Na					Biospecimen Core	Biomarkers of Cognitive Decline Among Normal Adults (BIOCARD) Study Team
Paul	Worley					Biospecimen Core	Biomarkers of Cognitive Decline Among Normal Adults (BIOCARD) Study Team
Ann	Ervin					Informatics Core	Biomarkers of Cognitive Decline Among Normal Adults (BIOCARD) Study Team
David	Shade					Informatics Core	Biomarkers of Cognitive Decline Among Normal Adults (BIOCARD) Study Team
Jennifer	Jones					Informatics Core	Biomarkers of Cognitive Decline Among Normal Adults (BIOCARD) Study Team
Hamadou	Coulibaly					Informatics Core	Biomarkers of Cognitive Decline Among Normal Adults (BIOCARD) Study Team
Mei-Cheng	Wang					Biostatistics Core	Biomarkers of Cognitive Decline Among Normal Adults (BIOCARD) Study Team
Daisy	Zhu					Biostatistics Core	Biomarkers of Cognitive Decline Among Normal Adults (BIOCARD) Study Team
Jiangxia	Wang					Biostatistics Core	Biomarkers of Cognitive Decline Among Normal Adults (BIOCARD) Study Team
Juan	Troncoso					Neuropathology Core	Biomarkers of Cognitive Decline Among Normal Adults (BIOCARD) Study Team
David	Nauen					Neuropathology Core	Biomarkers of Cognitive Decline Among Normal Adults (BIOCARD) Study Team
Kendra	Ellis					Neuropathology Core	Biomarkers of Cognitive Decline Among Normal Adults (BIOCARD) Study Team
Jonathan	Haines			Case Western Reserve University			Alzheimer's Disease Sequencing Project (ADSP)
Audrey	Lynn			Case Western Reserve University			Alzheimer's Disease Sequencing Project (ADSP)
Scott M	Williams			Case Western Reserve University			Alzheimer's Disease Sequencing Project (ADSP)
Nicolas R.	Wheeler			Case Western Reserve University			Alzheimer's Disease Sequencing Project (ADSP)
Alan J.	Lerner			Case Western Reserve University			Alzheimer's Disease Sequencing Project (ADSP)
Jackie	Bartlett			Case Western Reserve University			Alzheimer's Disease Sequencing Project (ADSP)
Penelope	Benchek			Case Western Reserve University			Alzheimer's Disease Sequencing Project (ADSP)
Yeunjoo	Song			Case Western Reserve University			Alzheimer's Disease Sequencing Project (ADSP)
Jungsoo	Gim			Chosun University, Korea			Alzheimer's Disease Sequencing Project (ADSP)
Sandra	Barral Rodriguez			Columbia University			Alzheimer's Disease Sequencing Project (ADSP)
Phil	De Jager			Columbia University			Alzheimer's Disease Sequencing Project (ADSP)
Suzanne	Leal			Columbia University			Alzheimer's Disease Sequencing Project (ADSP)
Yiyi	Ma			Columbia University			Alzheimer's Disease Sequencing Project (ADSP)
Richard	Mayeux			Columbia University			Alzheimer's Disease Sequencing Project (ADSP)
Christiane	Reitz			Columbia University			Alzheimer's Disease Sequencing Project (ADSP)
Dolly	Reyes-Dumeyer			Columbia University			Alzheimer's Disease Sequencing Project (ADSP)
Badri N.	Vardarajan			Columbia University			Alzheimer's Disease Sequencing Project (ADSP)
Shahzhad	Ahmad			Erasmus Medical University/Rotterdam			Alzheimer's Disease Sequencing Project (ADSP)
Hata	Comic			Erasmus Medical University/Rotterdam			Alzheimer's Disease Sequencing Project (ADSP)
Hannah	Craft			Indiana University/Indiana ADRC			Alzheimer's Disease Sequencing Project (ADSP)

*First Name and Middle Initial(s)	*Last Name	*Suffix (eg, Jr, III)	Academic Degrees	Institution	Location (city, state/province, country)	Role or Contribution, eg, chair, principal investigator	Group (if more than 1 Group listed in the byline) and/or Subgroup (eg, Steering Committee)
Taeho	Jo			Indiana University/Indiana ADRC			Alzheimer's Disease Sequencing Project (ADSP)
Kwangsik	Nho			Indiana University/Indiana ADRC			Alzheimer's Disease Sequencing Project (ADSP)
Shannon L	Risacher			Indiana University/Indiana ADRC			Alzheimer's Disease Sequencing Project (ADSP)
Andrew J	Saykin			Indiana University/Indiana ADRC			Alzheimer's Disease Sequencing Project (ADSP)
Min Soo	Byun			Seoul National University, Korea			Alzheimer's Disease Sequencing Project (ADSP)
Dong Young	Lee			Seoul National University, Korea			Alzheimer's Disease Sequencing Project (ADSP)
Dahyun	Yi			Seoul National University, Korea			Alzheimer's Disease Sequencing Project (ADSP)
John	McNeil			Monash University			Alzheimer's Disease Sequencing Project (ADSP)
Shea	Andrews			Mt. Sinai School of Medicine			Alzheimer's Disease Sequencing Project (ADSP)
Brian	Fulton-Howard			Mt. Sinai School of Medicine			Alzheimer's Disease Sequencing Project (ADSP)
Alison	Goate			Mt. Sinai School of Medicine			Alzheimer's Disease Sequencing Project (ADSP)
Jack	Humphrey			Mt. Sinai School of Medicine			Alzheimer's Disease Sequencing Project (ADSP)
Dado	Marcora			Mt. Sinai School of Medicine			Alzheimer's Disease Sequencing Project (ADSP)
Tulsi	Patel			Mt. Sinai School of Medicine			Alzheimer's Disease Sequencing Project (ADSP)
Towfique	Raj			Mt. Sinai School of Medicine			Alzheimer's Disease Sequencing Project (ADSP)
Alan	Renton			Mt. Sinai School of Medicine			Alzheimer's Disease Sequencing Project (ADSP)
Andrew	Sharp			Mt. Sinai School of Medicine			Alzheimer's Disease Sequencing Project (ADSP)
Ricardo	Vialle			Mt. Sinai School of Medicine			Alzheimer's Disease Sequencing Project (ADSP)
Kelley	Faber			NCRAD			Alzheimer's Disease Sequencing Project (ADSP)
Tatiana	Foroud			NCRAD			Alzheimer's Disease Sequencing Project (ADSP)
Kelly	Nudelman			NCRAD			Alzheimer's Disease Sequencing Project (ADSP)
Kaci	Lacy			NCRAD			Alzheimer's Disease Sequencing Project (ADSP)
David	Knowles			New York Genome Center & Columbia University			Alzheimer's Disease Sequencing Project (ADSP)
Chirag	Lakhani			New York Genome Center			Alzheimer's Disease Sequencing Project (ADSP)
Anjali	Das			New York Genome Center & Columbia University			Alzheimer's Disease Sequencing Project (ADSP)
Teresa	Lin			New York Genome Center			Alzheimer's Disease Sequencing Project (ADSP)
Yun	Freudenberg-Hua			Northwell Health			Alzheimer's Disease Sequencing Project (ADSP)
Min Soo	Byun			Seoul National University			Alzheimer's Disease Sequencing Project (ADSP)
Dong Young	Lee			Seoul National University			Alzheimer's Disease Sequencing Project (ADSP)
Sungho	Wang			Seoul National University			Alzheimer's Disease Sequencing Project (ADSP)
Dahyun	Yi			Seoul National University			Alzheimer's Disease Sequencing Project (ADSP)
Michael	Greicius			Stanford University			Alzheimer's Disease Sequencing Project (ADSP)
Anshul	Kundaje			Stanford University			Alzheimer's Disease Sequencing Project (ADSP)
Stephen	Montgomery			Stanford University			Alzheimer's Disease Sequencing Project (ADSP)
Tom	Montine			Stanford University			Alzheimer's Disease Sequencing Project (ADSP)
Ryan	Corces			University of California, San Francisco			Alzheimer's Disease Sequencing Project (ADSP)
Vilmundur	Guðnason			University of Iceland			Alzheimer's Disease Sequencing Project (ADSP)
Gary	Beecham			University of Miami			Alzheimer's Disease Sequencing Project (ADSP)
Mike	Cuccaro			University of Miami			Alzheimer's Disease Sequencing Project (ADSP)
Tony	Griswold			University of Miami			Alzheimer's Disease Sequencing Project (ADSP)
Kara	Hamilton-Nelson			University of Miami			Alzheimer's Disease Sequencing Project (ADSP)
Brian	Kunkle			University of Miami			Alzheimer's Disease Sequencing Project (ADSP)
Nicholas	Kushch			University of Miami			Alzheimer's Disease Sequencing Project (ADSP)

*First Name and Middle Initial(s)	*Last Name	*Suffix (eg, Jr, III)	Academic Degrees	Institution	Location (city, state/province, country)	Role or Contribution, eg, chair, principal investigator	Group (if more than 1 Group listed in the byline) and/or Subgroup (eg, Steering Committee)
Eden	Martin			University of Miami			Alzheimer's Disease Sequencing Project (ADSP)
Pedro	Mena			University of Miami			Alzheimer's Disease Sequencing Project (ADSP)
Peggy	Pericak-Vance			University of Miami			Alzheimer's Disease Sequencing Project (ADSP)
Farid	Rajabli			University of Miami			Alzheimer's Disease Sequencing Project (ADSP)
Mike	Schmidt			University of Miami			Alzheimer's Disease Sequencing Project (ADSP)
Susan	Slifer			University of Miami			Alzheimer's Disease Sequencing Project (ADSP)
Jeffery	Vance			University of Miami			Alzheimer's Disease Sequencing Project (ADSP)
Karen	Nuytemans			University of Miami			Alzheimer's Disease Sequencing Project (ADSP)
Patrice	Whitehead			University of Miami			Alzheimer's Disease Sequencing Project (ADSP)
Larry D	Adams			University of Miami			Alzheimer's Disease Sequencing Project (ADSP)
John R.	Gilbert			University of Miami			Alzheimer's Disease Sequencing Project (ADSP)
Sharon	Kardia			University of Michigan			Alzheimer's Disease Sequencing Project (ADSP)
Jennifer	Smith			University of Michigan			Alzheimer's Disease Sequencing Project (ADSP)
Wei	Zhao			University of Michigan			Alzheimer's Disease Sequencing Project (ADSP)
Najaf	Amin			University of Oxford			Alzheimer's Disease Sequencing Project (ADSP)
Cornelia	van Duijn			University of Oxford			Alzheimer's Disease Sequencing Project (ADSP)
Laura B	Cantwell			University of Pennsylvania			Alzheimer's Disease Sequencing Project (ADSP)
Yi-Fan	Chou			University of Pennsylvania			Alzheimer's Disease Sequencing Project (ADSP)
Christos	Davatzikos			University of Pennsylvania			Alzheimer's Disease Sequencing Project (ADSP)
Heather	Nicaretta			University of Pennsylvania			Alzheimer's Disease Sequencing Project (ADSP)
Amanda B.	Kuzma			University of Pennsylvania			Alzheimer's Disease Sequencing Project (ADSP)
Wan-Ping	Lee			University of Pennsylvania			Alzheimer's Disease Sequencing Project (ADSP)
Fanny	Leung			University of Pennsylvania			Alzheimer's Disease Sequencing Project (ADSP)
John	Malamon			University of Pennsylvania			Alzheimer's Disease Sequencing Project (ADSP)
Adam	Naj			University of Pennsylvania			Alzheimer's Disease Sequencing Project (ADSP)
Jerry	Schellenberg			University of Pennsylvania			Alzheimer's Disease Sequencing Project (ADSP)
Li	Shen			University of Pennsylvania			Alzheimer's Disease Sequencing Project (ADSP)
Li-San	Wang			University of Pennsylvania			Alzheimer's Disease Sequencing Project (ADSP)
Otto	Valladares			University of Pennsylvania			Alzheimer's Disease Sequencing Project (ADSP)
Alexis	Lerro Rose			University of Pennsylvania			Alzheimer's Disease Sequencing Project (ADSP)
Andy	Wilk			University of Pennsylvania			Alzheimer's Disease Sequencing Project (ADSP)
Beth	Dombrosk			University of Pennsylvania			Alzheimer's Disease Sequencing Project (ADSP)
Emily	Greenfest-Allen			University of Pennsylvania			Alzheimer's Disease Sequencing Project (ADSP)
Flawless	Robbins			University of Pennsylvania			Alzheimer's Disease Sequencing Project (ADSP)
Heather	White			University of Pennsylvania			Alzheimer's Disease Sequencing Project (ADSP)
Jake	Haut			University of Pennsylvania			Alzheimer's Disease Sequencing Project (ADSP)
Jascha	Brettschneider			University of Pennsylvania			Alzheimer's Disease Sequencing Project (ADSP)
Jeffrey	Cifello			University of Pennsylvania			Alzheimer's Disease Sequencing Project (ADSP)
Jin	Sha			University of Pennsylvania			Alzheimer's Disease Sequencing Project (ADSP)
Joseph	Manuel			University of Pennsylvania			Alzheimer's Disease Sequencing Project (ADSP)
Lauren	Bass			University of Pennsylvania			Alzheimer's Disease Sequencing Project (ADSP)
Liming	Qu			University of Pennsylvania			Alzheimer's Disease Sequencing Project (ADSP)
Luke	Carter			University of Pennsylvania			Alzheimer's Disease Sequencing Project (ADSP)
Maureen	Kirsch			University of Pennsylvania			Alzheimer's Disease Sequencing Project (ADSP)
Michelle K	Moon			University of Pennsylvania			Alzheimer's Disease Sequencing Project (ADSP)
Naveen	Saravanan			University of Pennsylvania			Alzheimer's Disease Sequencing Project (ADSP)

*First Name and Middle Initial(s)	*Last Name	*Suffix (eg, Jr, III)	Academic Degrees	Institution	Location (city, state/province, country)	Role or Contribution, eg, chair, principal investigator	Group (if more than 1 Group listed in the byline) and/or Subgroup (eg, Steering Committee)
Pavel	Kuksa			University of Pennsylvania			Alzheimer's Disease Sequencing Project (ADSP)
Pei-Chuan	Ho			University of Pennsylvania			Alzheimer's Disease Sequencing Project (ADSP)
Peter	Keskinen			University of Pennsylvania			Alzheimer's Disease Sequencing Project (ADSP)
PK	Gangadharan			University of Pennsylvania			Alzheimer's Disease Sequencing Project (ADSP)
Sam	Tate			University of Pennsylvania			Alzheimer's Disease Sequencing Project (ADSP)
Taha	Iqbal			University of Pennsylvania			Alzheimer's Disease Sequencing Project (ADSP)
Shaney	Chuang			University of Pennsylvania			Alzheimer's Disease Sequencing Project (ADSP)
Wenhwai	Horng			University of Pennsylvania			Alzheimer's Disease Sequencing Project (ADSP)
Yi	Zhao			University of Pennsylvania			Alzheimer's Disease Sequencing Project (ADSP)
Youli	Ren			University of Pennsylvania			Alzheimer's Disease Sequencing Project (ADSP)
Jimmy	Jin			University of Pennsylvania			Alzheimer's Disease Sequencing Project (ADSP)
Zile	Katanic			University of Pennsylvania			Alzheimer's Disease Sequencing Project (ADSP)
Robert Babak	Frayabi			University of Pennsylvania			Alzheimer's Disease Sequencing Project (ADSP)
Mingyao	Li			University of Pennsylvania			Alzheimer's Disease Sequencing Project (ADSP)
Marissa	Cranney			University of Pennsylvania			Alzheimer's Disease Sequencing Project (ADSP)
Kamnaa	Arya			University of Pennsylvania			Alzheimer's Disease Sequencing Project (ADSP)
Heng	Huang			University of Pittsburgh			Alzheimer's Disease Sequencing Project (ADSP)
Sophia	Thomopolous			University of Southern California			Alzheimer's Disease Sequencing Project (ADSP)
Paul	Thompson			University of Southern California			Alzheimer's Disease Sequencing Project (ADSP)
Myriam	Fornage			University of Texas Houston			Alzheimer's Disease Sequencing Project (ADSP)
Chloe	Sarnowski			University of Texas Houston			Alzheimer's Disease Sequencing Project (ADSP)
Peter	St. George-Hyslop			University of Toronto			Alzheimer's Disease Sequencing Project (ADSP)
Josh	Bis			University of Washington			Alzheimer's Disease Sequencing Project (ADSP)
Liz	Blue			University of Washington			Alzheimer's Disease Sequencing Project (ADSP)
Andrea	Horimoto			University of Washington			Alzheimer's Disease Sequencing Project (ADSP)
Rafael	Nafikov			University of Washington			Alzheimer's Disease Sequencing Project (ADSP)
Tim	Thornton			University of Washington			Alzheimer's Disease Sequencing Project (ADSP)
Ellen	Wijsman			University of Washington			Alzheimer's Disease Sequencing Project (ADSP)
Seth	Temple			University of Washington			Alzheimer's Disease Sequencing Project (ADSP)
Tyler	Day			University of Washington			Alzheimer's Disease Sequencing Project (ADSP)
Eugene	Lin			University of Washington			Alzheimer's Disease Sequencing Project (ADSP)
Bruce	Psaty			University of Washington			Alzheimer's Disease Sequencing Project (ADSP)
Dagmar	Bacikova			USUHS			Alzheimer's Disease Sequencing Project (ADSP)
Clifton	Dalgard			USUHS			Alzheimer's Disease Sequencing Project (ADSP)
Bernard	Fongang			UT Health San Antonio			Alzheimer's Disease Sequencing Project (ADSP)
Xueqiu	Jian			UT Health San Antonio			Alzheimer's Disease Sequencing Project (ADSP)
Muralidharan	Sargurupremraj			UT Health San Antonio			Alzheimer's Disease Sequencing Project (ADSP)
Claudia	Satizabal			UT Health San Antonio			Alzheimer's Disease Sequencing Project (ADSP)
Sudha	Seshadri			UT Health San Antonio			Alzheimer's Disease Sequencing Project (ADSP)
Habil	Zare			UT Health San Antonio			Alzheimer's Disease Sequencing Project (ADSP)
Jose	Bras			Van Andel Institute			Alzheimer's Disease Sequencing Project (ADSP)
Rita	Guerreiro			Van Andel Institute			Alzheimer's Disease Sequencing Project (ADSP)
Piper	Below			Vanderbilt University			Alzheimer's Disease Sequencing Project (ADSP)
Logan	Dumitrescu			Vanderbilt University			Alzheimer's Disease Sequencing Project (ADSP)
Timothy	Hohman			Vanderbilt University			Alzheimer's Disease Sequencing Project (ADSP)
Carlos	Cruchaga			Washington University St Louis			Alzheimer's Disease Sequencing Project (ADSP)

Supplemental Online Content: Nonauthor Collaborators
*First name, last name, and suffix (if applicable) are required and will appear in PubMed.

*First Name and Middle Initial(s)	*Last Name	*Suffix (eg, Jr, III)	Academic Degrees	Institution	Location (city, state/province, country)	Role or Contribution, eg, chair, principal investigator	Group (if more than 1 Group listed in the byline) and/or Subgroup (eg, Steering Committee)
Susan	Dutcher			Washington University St Louis			Alzheimer's Disease Sequencing Project (ADSP)
Victoria	Fernandez			Washington University St Louis			Alzheimer's Disease Sequencing Project (ADSP)
Achal	Neupane			Washington University St Louis			Alzheimer's Disease Sequencing Project (ADSP)
Jung-Ying	Tzeng			NC State University			Alzheimer's Disease Sequencing Project (ADSP)
Shin-Yi	Chou			Lehigh University			Alzheimer's Disease Sequencing Project (ADSP)
Alessandra F.A.	Chesi			Childrens Hospital of Pennsylvania			Alzheimer's Disease Sequencing Project (ADSP)
Struan F.A.	Grant			Childrens Hospital of Pennsylvania			Alzheimer's Disease Sequencing Project (ADSP)
