# Supplementary material for: White Matter Abnormalities and Cognition in Aging and Alzheimer Disease
Source: JAMA Neurol. 2025 Jun 9;82(8):825–36. doi: 10.1001/jamaneurol.2025.1601 (PMC12150229; doi:10.1001/jamaneurol.2025.1601)
Supplement: Supplement 4. — Data Sharing Statement [file jamaneurol-e251601-s004.pdf]

Data Sharing Statement

Peter. White Matter Abnormalities and Cognition in Aging and Alzheimer Disease. *JAMA Neurol*. Published June 09, 2025. doi:10.1001/jamaneurol.2025.1601

Data
Data available: No

Additional Information
Explanation for why data not available: We are not providing data as this is already a secondary data study. Original data can be obtained from the original studies.
